# Fully Automated Segmentation of the Pons and Midbrain Using Human T1 MR Brain Images

**DOI:** 10.1371/journal.pone.0085618

**Published:** 2014-01-28

**Authors:** Salvatore Nigro, Antonio Cerasa, Giancarlo Zito, Paolo Perrotta, Francesco Chiaravalloti, Giulia Donzuso, Franceso Fera, Eleonora Bilotta, Pietro Pantano, Aldo Quattrone, the Alzheimer's Disease Neuroimaging Initiative

**Affiliations:** 1 Institute of Bioimaging and Molecular Physiology, National Research Council, Catanzaro, Italy; 2 Institute of Neurology, University “Magna Graecia”, Catanzaro, Italy; 3 Laboratory of Electrophysiology for Translational Neuroscience, National Research Council, Rome, Italy; 4 Department of Clinical Neuroscience, ‘S. Giovanni Calibita’ Fatebenefratelli Hospital, Rome, Italy; 5 Evolutionary Systems Group, University of Calabria, Cosenza, Italy; 6 Department of Medical and Surgical Sciences, University “Magna Graecia”, Catanzaro, Italy; INSERM, Paris, France

## Abstract

**Purpose:**

This paper describes a novel method to automatically segment the human brainstem into midbrain and pons, called LABS: *Landmark-based Automated Brainstem Segmentation*. LABS processes high-resolution structural magnetic resonance images (MRIs) according to a revised landmark-based approach integrated with a thresholding method, without manual interaction.

**Methods:**

This method was first tested on morphological T1-weighted MRIs of 30 healthy subjects. Its reliability was further confirmed by including neurological patients (with Alzheimer's Disease) from the ADNI repository, in whom the presence of volumetric loss within the brainstem had been previously described. Segmentation accuracies were evaluated against expert-drawn manual delineation. To evaluate the quality of LABS segmentation we used volumetric, spatial overlap and distance-based metrics.

**Results:**

The comparison between the quantitative measurements provided by LABS against manual segmentations revealed excellent results in healthy controls when considering either the midbrain (DICE measures higher that 0.9; Volume ratio around 1 and Hausdorff distance around 3) or the pons (DICE measures around 0.93; Volume ratio ranging 1.024–1.05 and Hausdorff distance around 2). Similar performances were detected for AD patients considering segmentation of the pons (DICE measures higher that 0.93; Volume ratio ranging from 0.97–0.98 and Hausdorff distance ranging 1.07–1.33), while LABS performed lower for the midbrain (DICE measures ranging 0.86–0.88; Volume ratio around 0.95 and Hausdorff distance ranging 1.71–2.15).

**Conclusions:**

Our study represents the first attempt to validate a new fully automated method for *in vivo* segmentation of two anatomically complex brainstem subregions. We retain that our method might represent a useful tool for future applications in clinical practice.

## Introduction

In neuroimaging, brain segmentation plays an important role in several medical applications. This field of study has attracted much interest from the clinical community since *in vivo* automatic quantification of anatomical abnormalities in critical brain regions represents crucial information that might significantly impact clinical management and practice (i.e. identification of new biomarkers).

Although manual segmentation is currently considered the gold standard approach to determine the morphology of brain regions [1], this method is traditionally time-consuming and dependent on rater experience. Designing algorithms that automatically segment brain regions is challenging, especially for highly variable structures such as the ones located at subcortical level. In the last few years, a large amount of robust and fully automated segmentation tools have been developed for extracting complex anatomical brain regions. To overall summarize these methods several theoretical categories have been proposed.

For instance, automated segmentation methods may be classified in three broad classes:

- The first class of methods achieves brain tissue segmentation by applying statistical classification methods to the signal intensities. The initial works used information from only one MRI contrast (i.e., T1 weighted images) [2]–[4] while advanced techniques employed very complicated adaptive segmentation algorithms [5] or multichannel tissue segmentation methods combining data from different imaging contrasts [6], which drastically improved the ability to segment subcortical structures. However, analysis of the signal intensity alone is not sufficient to distinguish between different subcortical gray mater structures. For this reason, it has been suggested [7], [8] that incorporating *a priori* constraints, such as corresponding anatomical landmarks, can improve the identification of boundaries in regions where anatomical boundaries are fuzzy and greater residual anatomic variability remains.
- The second class refers to methods that rely primarily on strong shape, mathematical models, where the algorithms are dedicated to discriminate between brain regions by using morphological contents (geometry) of MRIs. For example, FreeSurfer [9] employs affine transformations while combining the voxel intensity values relative to a probability distribution for tissue classes, with the information of the voxel location in respect with the neighboring structures obtained from a manually labeled atlas. Again, Pohl et al. [10] used an Expectation Maximization (EM) type algorithm with shape priors to perform segmentation. The EM algorithm is a general method of finding the maximum likelihood estimate of the parameters of an underlying distribution from a given data set when the data is incomplete or has missing values. This algorithm has been employed by several 3D automatic tools for improving brain segmentation [7], [11].
- Finally there are others tools that use mathematical models for brain segmentation that include both the appearance (voxel intensity) and shape (geometry) of each single brain region [12], [13]. For example, in FSL/FIRST [13], automated segmentations proceed via a Bayesian probabilistic approach using shape and appearance models, built from a library of manually segmented images, parameterized as surface meshes and then modeled as point distributions. Using the learned models, FIRST searches through linear combinations of shape modes of variation (principal components) to find the most probable shape instance given the observed intensities from the input image. FIRST uses an empirically determined fixed number of modes (iterations) for each structure. Finally, the vertex information or models are transferred back to the native space in which the boundaries were corrected and volumes (labels) were generated.

Again, an elegant division proposed by Khan et al. [14] distinguishes these methods in:

- *Knowledge-driven methods*, which use implicit or explicit anatomical knowledge to guide the segmentation, mainly for individual structure such as caudate nucleus and hippocampus/amygdala [15], [16].
- *Probabilistic-based methods*, which treat segmentation as a classification problem and estimate the labeling that maximizes an *a posteriori* probability given specific constraints (such as Freesufer [9]).
- *Deformable template-based methods*, which involve finding a geometric transformation from a pre-labeled template scan to the target scan and propagating the labels with the same transformation to label the target brain.

Although all these methods contributed towards quickly and accurately obtaining *in vivo* 3D volumetric quantification of almost all clinically relevant subcortical brain regions, there is a specific subcortical region of the human brain that still remains sparsely studied: the brainstem. Among the above-mentioned techniques only few methods directly provide an objective quantification of the brainstem [6], [9], [13]. The human brainstem is a very complex structure composed by two sub-regions of great clinical interest, the pons and the midbrain, far from being adequately segmented. The automatic 3D segmentation of these two regions is essential by virtue of their neurophysiological peculiarities. The midbrain holds several important nuclei, such as substantia nigra and red nucleus, involved in several neurophysiological processes regulating emotion and motor behaviors. The pons connects the cerebellum to the main portion of the brain through two thick structures known as cerebellar peduncles. White matter inside the pons is crucial to a number of important motor functions, including: arousal, sleeping and sensory awareness. Moreover, there is increasing evidence that some neurodegenerative diseases, such as Parkinson's disease (PD) and Alzheimer's disease (AD), are characterized by early (and distinct) involvement of these two regions [17]–[22]. At this time, the only validated MRI-based measurement employed in clinical practice derives from conventional MRI using manual morphometric quantification. In fact, several authors [19], [22], [23], using different approaches, demonstrated that the diameter or area assessed on the mid-sagittal plane of these two brainstem structures, allow a reliable differential diagnosis of PD with respect to parkinsonism of different etiology, such as Progressive Supranuclear Palsy (PSP)(clinically similar to PD patients, but with a more rapid disease progression). In all these studies, the steps to identify and quantify the pons and midbrain are based upon manual intervention, thus making it difficult to assess reliability between the methods and the application on large samples (due to time constraints). For this reason, an accurate *in vivo* automatic measurement of these two regions is an essential step for improving clinical management of neurological patients, as well as, in longitudinal and prospective studies, thereby eliminating the problems associated with manual segmentation.

In this paper we present a novel method able to perform an unbiased automatic segmentation of the pons and the midbrain using high-resolution structural MRIs, in order to obtain accurate measurements of these two anatomically complex subcortical regions. This method is called: LABS (*Landmark-based Automated Brainstem Segmentation*). LABS is based on a revised landmark-based approach using general, widely-accepted knowledge of human brain morphology, which integrates information about tissue class, structure and position, without requiring manual intervention. This information is integrated with a “thresholding-based approach”, that allows separation of the two classes through the choice of a pixel intensity value (“threshold”) [24], so that all pixels with intensity greater than the threshold are grouped within a class and all other pixels into another class. Our method has been tested using a 3Tesla MRI scanner on healthy populations having a large age-range and validated using quantitative comparisons. Moreover, to further validate our tool in the neurological realm, we also tested automatic 3D segmentation on patients with AD, obtained from another MRI scanner (Siemens 1.5T, ADNI database)

## Methods

### Participants

Thirty right-handed healthy right-handed subjects (mean age: 42.7±2.5 years; age range from 24 to 71 y; 15 males) participated in this study. Careful screening was performed to ensure that participants were free of psychiatric and/or neurological history, psychoactive drug treatment, drug or alcohol abuse, and had no MR contraindications. All participants gave written informed consent to participate in the present study, approved by the Ethical Committee of the University ‘Magna Graecia’ of Catanzaro according to the declaration of Helsinki.

To further validate our study, we enrolled 40 patients with AD (mean age: 71.8±6.8 years; 22 males) and 40 age-/sex-matched healthy controls (mean age: 73.7±10.1 years; 23 males) from the Alzheimer's disease Neuroimaging Initiative (ADNI) repository (www.loni.ucla.edu/ADNI) (Table S1 and Table S2). The ADNI project was launched in 2003 by the National Institute on Aging (NIA), the National Institute of Biomedical Imaging and Bioengineering (NIBIB), the Food and Drug Administration (FDA), together with private pharmaceutical companies and non-profit organizations. The primary goal of ADNI has been to test whether serial MRIs, positron emission tomography, several biological markers, and clinical/neuropsychological assessments can be suitably combined to measure the progression of mild cognitive impairment and early AD.

### MRI Acquisition

Brain MRI for healthy controls was performed on a 3 Tesla scanner with an 8-channel head coil (Discovery MR-750, GE, Milwaukee, WI, USA) at the Neuroimaging Research Unit, Institute of Neurological Sciences, National Research Council, Catanzaro, Italy. Structural MRI data were acquired using a 3D T1-weighted spoiled gradient echo (SPGR) sequence with the following parameters: TR: 3.7 ms, TE: 9.2 ms, FOV: 25.6 mm, flip angle 12°, voxel-size 1×1×1 mm^3^, producing 368 slices covering the entire brain. Subjects were positioned to lie comfortably in the scanner with a forehead-restraining strap and various foam pads to ensure head fixation. All scans had equally good quality with negligible motion artifacts.

AD patients and age-/sex-matched healthy controls followed the ADNI MRI acquisition protocol [25]. We only used images acquired with 1.5 T scanners and already pre-processed to avoid artifacts due to magnetic field inhomogeneity and signal drifts [25]. For each subject, we used the MRI considered as the “best” quality scan by the ADNI investigators. In the description of the ADNI methods (http://www.loni.ucla.edu/ADNI/Data/ADNI_Data.shtml), the “best” quality image is the one which was used for the complete pre-processing steps. The identification numbers of the images used in this study are reported in Table S1 and Table S2.

### Algorithm

**Figure 1:**
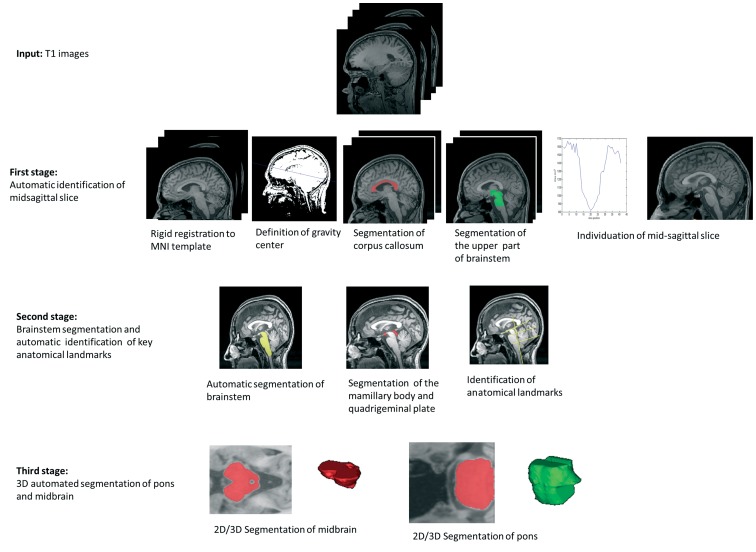
Stages of algorithm.

In this section we describe the proposed automated segmentation approach to define the pons and midbrain parcellation. This method consists of three stages summarized by the outline in the Figure 1, whose details will be presented in the following sections. The input data for our method consisted of high-resolution T1-weighted structural MRI scans. This method was first validated on population of 30 right-handed healthy subjects enrolled in our institute. Next, we tested its robustness on additional cohorts of AD and controls extracted from ADNI database.

The first stage was aimed to automatically define the mid-sagittal plane. For this reason, morphological high-resolution T1-weighted images were rigidly registered (using a 6-parameter affine registration) to the template MNI image based on the mutual information metric using the software SPM8 software (http://www.fil.ion.ucl.ac.uk/spm) and resampling the registered T1 using cubic spline interpolation. Subsequently, we individuated the mid-sagittal plane using specific anatomical landmarks such as the corpus callosum and the upper part of the brainstem. In the second stage we segmented the brainstem, mammillary body and the quadrigeminal plate using the mid-sagittal plane. The outline of these latter subcortical structures is extremely important for defining the planes useful for dividing brainstem into the pons and midbrain, following previous radiological and anatomical criteria [22], [26]. Finally, in the last stage we used the planes of cut, previously defined, to delineate two subvolumes of images in which we may respectively segment and extract the entire volumes of the pons and midbrain.

Each step of our method is based upon a combination of a revised landmark-based approach together with a threshold-based algorithm. The threshold value used for each step was obtained using a revised Otsu et al. [24] approach, introducing some correction factors to improve the quality of the segmentation.

### Individuation of mid-sagittal plane

The first stage was characterized by the individuation of the mid-sagittal plane within the 368 slices of our morphological T1-weighted sequence. In general, the mid-sagittal plane can be defined as either the plane best matching the cerebral interhemispheric fissure [27], [28] or the plane maximizing the bilateral symmetry [29]–[31]. A large amount of work has been dedicated to the automatic individuation of this plane using different approaches [32]–[36].

In our method we registered the volume of images to a template MNI using a rigid transformation. Next, to reach automatic identification of the “mid-sagittal” plane, several pre-processing steps were needed. First, we individuated in the overall T1-weighted sequence, a sub-volume of 40 images, called S^1^, (arbitrarily) centered on the “middle slice” of slice number 184. Then, we defined the gravity center of the headmask on the middle slice. In particular, the gravity center was obtained by thresholding the morphological T1-weighted images using Otsu's method [24] (Figure S1). In this way we obtained a binary mask where the coordinates X and Y of the barycenter B were given by Eq. 1, 2:
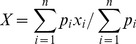
(1)

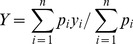
(2)where (x_i_,y_i_) and *p_i_* are the coordinates and the value of the i-th pixel and *n* is the numbers of pixels of image.

Second, knowing the position of the gravity center on the middle slice we may automatically segment the corpus callosum on each slice within S^1^. To do this we binarized the image and then we individuated the corpus callosum as the biggest connected component [37] that is positioned closer to the gravity center of the headmask. The threshold value (t) used to determine the connected component was different from that used to find the center of gravity of the head. In fact, we used a higher threshold in order to separate the corpus callosum, a structure with a very high intensity and therefore very clear in a T1-weighted sequence, from surrounding regions. The threshold value used for the segmentation of the corpus callosum was (Eq.3):
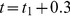
(3)where t1 was the threshold obtained using Otsu's binarization [24]. To determine the connected components, we used a neighborhood of eight pixels; and before the binarization, we enhanced the contrast of the grayscale image by transforming the values using contrast-limited adaptive histogram equalization (CLAHE) with a 0.02 contrast enhancement limit of 64 tiles [38].We were then able to automatically discriminate the corpus callosum on each single slice. Examples of automatic segmentation of the corpus callosum on different slices, included in the subvolume S^1^, have been reported in Figure S2.

**Figure 2:**
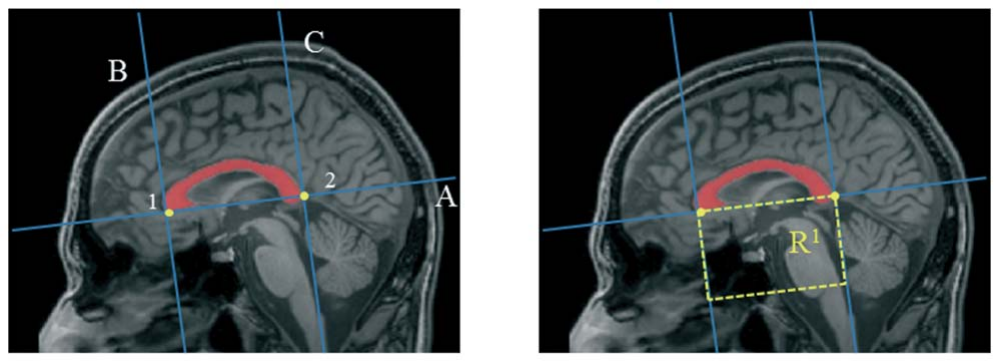
Definition of the R^1^ region used to segment the upper part of brainstem.

Considering the slice where we detected the smallest area of the corpus callosum, we automatically individuated three lines as shown in Figure 2. Line A is aligned with the left-bottom extreme point (point 1) and the right-bottom extreme point (point 2) of the corpus callosum. Lines B and C were perpendicular to the first line, and they crossed the corpus callosum respectively at points 1 and 2. These lines are helpful to discriminate the R^1^ region on each slice that will be used to segment the upper part of the brainstem. The dimensions of the rectangle R^1^ are L×L*2/3 where L was the distance between points 1 and 2.

Each slice within S^1^ was binarized and the pixels that are out of the rectangle R^1^ were set to zero. Next, we individuated the biggest connected component. This component is considered as the upper part of the brainstem (see Figure S3) and will be used to calculate the number of pixels composing it on each slice (quantification of area).

In accordance with morphological knowledge, we defined the “mid-sagittal plane” as the slice where the upper part of the brainstem is minimal. In this slice we have the biggest distance between the midbrain tectum and the quadrigeminal plate; consequently, we may observe the maximal expansion of the Sylvius aqueduct. In fact, the expansion of Sylvius aqueduct (see Figure S4) is considered an additional anatomical marker characterizing the mid-sagittal plane.

Calculating the area in each single slice included in the subvolume R^1^ (see Figure S5), the slice n° 20 is where we detected the minimal area of the upper part of the brainstem, thus it was definitively targeted as the mid-sagittal slice for this subject. Due to the extreme anatomical variability of the human brain, this latter step was repeated for each single subject. This approach has been further validated comparing the mid-sagittal slice, as automatically defined by LABS for each single subject, with that provided by two blind expert neuroradiologists (P.P, F.F). An excellent agreement was found. In fact, in 94% of the cases the algorithm individuated the same slice as those chosen by the neuroradiologists. Furthermore, in the remaining 6% of the cases, the difference in the spatial position between the automated and manual slice was minimal (about 1–2 slices).

### Individuation of boundaries of pons and midbrain on mid-sagittal slice

**Figure 3:**
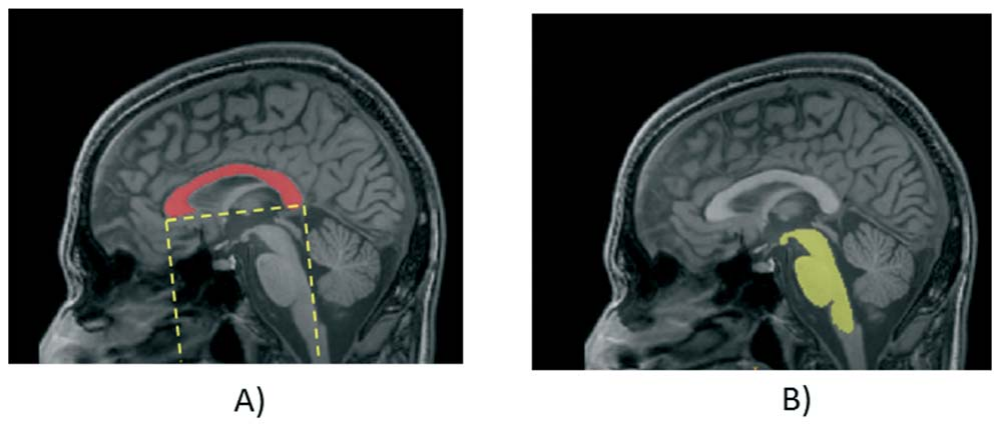
Segmentation of brainstem on mid-sagittal slice: a) identification of subregion of interest; b) segmentation of the entire brainstem using a threshold-based approach.

The manual approach proposed by Oba et al. [22], and Luft et al. [26], to identify the boundaries of the pons and the midbrain requires localizing the entire brainstem on the mid-sagittal slice. We used the same method shown in the above section. In particular, repeating some steps previously described, we identified the position of the corpus callosum on the mid-sagittal slice where we re-applied the R^1^ region (Figure 3A). Within this subvolume we individuated the brainstem as the largest connected component. In this case we did not define a lower limit for the rectangle R^1^ (as previously done, see Figure 2), thus allowing us to entirely segment the brainstem (Figure 3B).

The threshold value used to evaluate the connected component was (Eq.4):
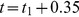
(4)


**Figure 4:**
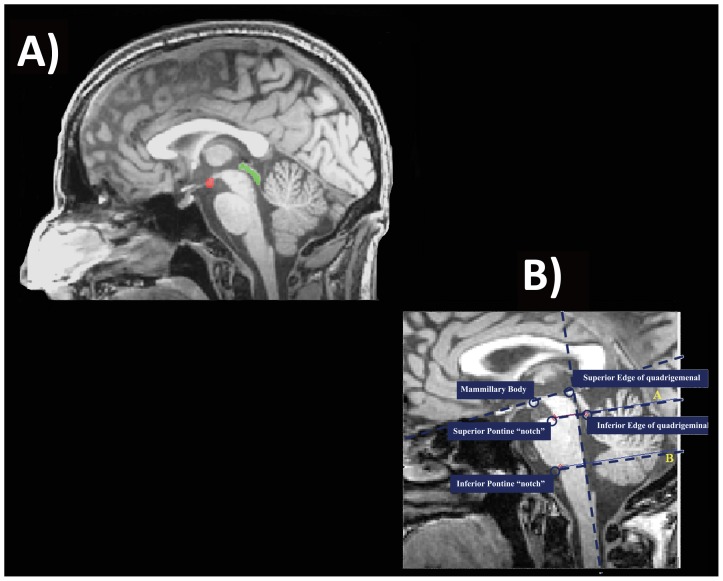
Identification of additional anatomical landmarks: a) mammillary body (red) and quadrigeminal plate (green); b) Boundaries of pons and midbrain on mid-sagittal slice according to the method proposed by Oba et al. [22] and Luft et al. [26].

After the segmentation of the brainstem (Figure 3B), we were able to identify another two critical anatomical landmarks that will become important for the following steps: mammillary body and quadrigeminal plate (Figure 4A). To identify the mammillary body we extracted the contour of the brainstem and studying the variation of thickness of the upper left profile we identified the enlargement of the rostral midbrain and so the mammillary body (Figure S6). To identify the quadrigeminal plate we employed again the R^1^ region, deleting the pixels belonging to the brainstem and identifying the plate as the connected component that had the center of gravity closer to the midbrain tectum (Figure S7).

At this point we can define the boundaries of midbrain and pons according to the method proposed by Oba et al. [22], and Luft et al., [26]. In particular the cranial border of the midbrain is defined as the axial plane through the mammillary body and the superior edge of the quadrigeminal plate. The caudal border is defined as the axial plane aligned for the superior pontine notch and the inferior edge of the quadrigeminal plate. The inferior boundary of pons is composed of a plane parallel to the latter plane and aligned with the inferior pontine notch (Figure 4B)

**Figure 5:**
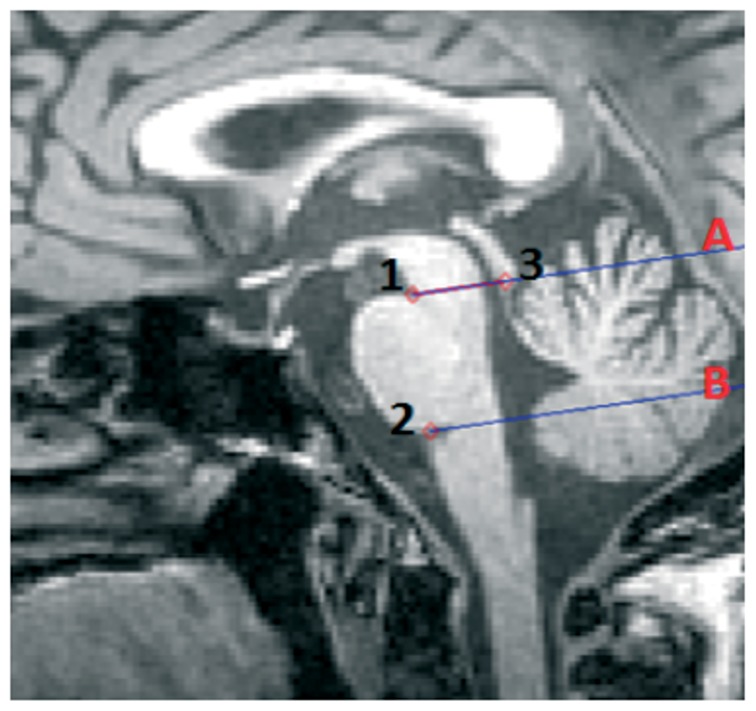
The Oba's method [22] to separate midbrain and pons on mid-sagittal slice. A first line passing through the superior pontine notch (point 1) and the inferior edge of the quadrigeminal plate (point 3) is drawn (line A) to separate midbrain and pons. A second line parallel to the first line passing through the inferior pontine notch (point 2) is drawn to define the inferior limit of the pons (line B).

To automatically define lines A and B, we employed three points (Figure 5). The positions of the first and the second points are determined by extrapolating the contour of the left side of the region and defining the variations of its profile. Points 1 and 2 represent respectively the beginning and the end of the anterior “hump” of the pons. To find the third point we isolated the quadrigeminal plate and defined its inferior point. Finally, we separated the pons from the cerebellum using a coronal plane through two points belonging to the dorsum of the brainstem (Figure S8).

### 3D segmentation of the pons and midbrain

In the final stage we extracted the entire volume of the pons and midbrain. First, we removed all pixels outside the brainstem setting the pixels to zero outside the boundaries defined in the previous sections and we resampled volumes to isotropic dimensions of 0.5 mm to allow a better spatial resolution of small brain structures. Then, using a thresholding approach we identified the area of the midbrain and pons on each slice, taking the largest connected component in the binarized image. Before the binarization we processed the image using contrast-limited adaptive histogram equalization. Furthermore, we re-introduced a corrector factor to binarize the image: the threshold value used was (Eq.5):
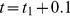
(5)


In the Figure S9 and S10 we highlighted in red the automatic segmentation of the midbrain and pons as performed by LABS. As shown in the last slices of Figure S10, the loss of anatomical definition in the upper part of the pons is dependent upon the complex separation between the pons and the middle cerebellar peduncles. In the neuroradiological and neuroimaging community there is no consensus on which anatomical landmark might be useful to improve this separation. For this reason, we decided to introduce another arbitrary landmark in order to better delineate the middle cerebellar peduncles. To do that, the lateral margins of the superior cerebellar peduncle were used for demarcating the boundaries between the middle cerebellar peduncles and pons. In particular, a volumetric slab of 40 mm (0.5-mm section thickness) tangent to the floor of the fourth ventricle was placed on a mid-sagittal plane to cover the entire extension of superior cerebellar peduncles (Figure S11).

The resulting oblique coronal images were used for subsequent individuation of two bilateral points employed as anatomical landmarks of the middle cerebellar peduncles (Figure S12). We used these two points to define two vertical lines, which we then used to separate the pons from the middle cerebellar peduncle (Figure S13).

**Figure 6:**
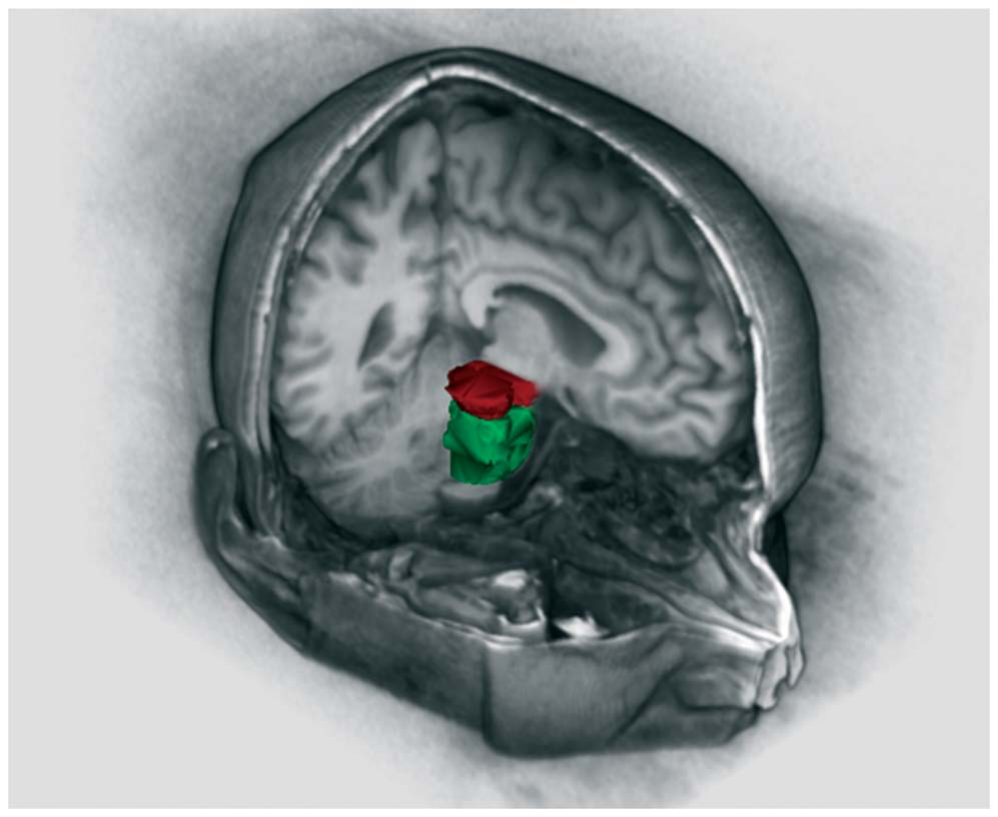
3D segmentation of pons and midbrain by LABS.

Finally, it is possible to obtain a 3D reconstruction of the automatic segmentation of the pons and midbrain using the matlab function, called “*isosurface*” (Figure 6). Overall, our algorithm run-time is between 10 min and 15 min on a 2.30 GHz Asus with an Intel Core i7-3610QM processor.

### MRI-based Manual Volume

Automated brainstem subregion accuracies were compared with manual segmentations as performed by two independent raters (G.Z)(F.F.), with more than 10 years of experience in neuroradiology, blind to the aim of this study. Manual segmentation was performed using MRIcro software (www.mricro.com), drawing, on the mid-sagittal slice, three lines that defined the cranial and the caudal borders of the midbrain and the inferior boundary of the pons in accordance with the methods described by Oba et al. [22] and Luft et al. [26]. All pixels outside these boundaries were then automatically set to zero and the data sets were reoriented into the axial plane. Raters were thus able to segment the pons and midbrain on the same slices used by LABS. The binary masks representing the results of manual segmentation were generated for each subject and considered as the gold standard to evaluate the performance of the LABS. The LABS output was similarly converted into binary images. Finally, the accuracy of LABS's performance was compared to manual tracing using the following criteria [39]: (i) percent volume overlap or Dice's coefficient as defined in Eq. 6 [40], (ii) percent volume difference as defined in Eq. 7 and (iii) the Hausdorff distance [41].

(i) The DICE coefficient is one of a number of measurements made to determine the extent of spatial overlap between two binary images. It is commonly used in the neuroimaging community to report on the performance of segmentation methods, and its values range between 0 (no overlap) and 1 (perfect agreement). Given two different labels of a structure, 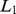 and 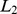, and a function V(L), which takes a label and returns its volume, the percent volume overlap is given by:
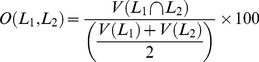
(6)


(ii) For each individual segmentation result we find the volume (V) as the number of labeled voxels multiplied by the voxel dimensions. We then calculate the percentage absolute volumetric difference (AVD) as the ratio of the absolute difference between the original volume and the segmented volume, to the original volume (Eq.7). The absolute value is used to account for some segmentation results having a lower volume than the gold standard, and others having a higher volume.
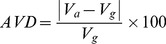
(7)


(iii) The modified Haussdorf distance between two point sets 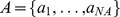
nd 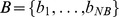
f size *NA* and *NB* is defined as (Eq. (8))
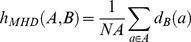
(8)where 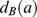 represents the minimum distance value at point 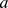 to the point set 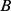.

### Statistical Analysis

Statistical analysis was performed with Statistical Package for Social Sciences software-SPSS (version 12.0, Chicago IL, USA). Assumptions for normality were tested for all continuous variables. Normality was tested using the Kolmogorov–Smirnov test. All variables were normally distributed.

To analyse effects of age, a linear regression model (*r*'s Pearson) was applied with the volumes of the midbrain and pons as extracted by LABS and by the two independent raters. Unpaired *t*-test was used to assess the presence of anatomical differences between the brainstem regions of healthy controls and AD patients. Age and gender were included in this model as nuisance variables. All statistical analyses had a two-tailed α level of <0.05 for defining significance.

## Results

### LABS validation on healthy controls

LABS showed that the average volumes of midbrain and pons in the human brain were of: 4031, 6 mm^3^ and 10440,4 mm^3^ (respectively, see Table 1 and Table 2) in agreement with previous post-mortem evidence [42]. The linear regression model revealed significant associations between age and the volumes of brainstem subregions either as measured by LABS (*r* = −0.44, *p*-value = 0.01; *r* = −0.36, *p*-value = 0.02; respectively for midbrain and pons) or by manual tracers (first rater: *r* = −0.36, *p*-value = 0.03; *r* = −0.31, *p*-value = 0.04; second rater: *r* = −0.34, *p*-value = 0.04; *r* = −0.33, *p*-value = 0.03; respectively for midbrain and pons).

**Table 1:** LABS performance on healthy controls: first rater.

*First Rater*	MIDBRAIN					PONS				
Healthy Controls	Manual Volume	LABS Volume	DICE	Hausdorff distance	Volume Ratio	Manual Volume	LABS Volume	DICE	Hausdorff distance	Volume Ratio
HC1	5256	4981	0.935	2.23	0.948	10178	10248	0.956	0.9054	1,007
HC2	3608	3380	0.943	2.96	0.937	10144	10341	0.940	1.525	1,019
HC3	4377	3899	0.927	2.99	0.891	10160	10701	0.923	1.5776	1,053
HC4	4182	3998	0.939	2.64	0.956	9238	9532	0.943	1.323	1,032
HC5	3314	3153	0.927	2.76	0.951	8875	9284	0.940	1.9243	1,046
HC6	5172	4922	0.927	2.53	0.952	10795	11351	0.931	1.7847	1,052
HC7	3864	3636	0.942	2.57	0.941	8934	9935	0.934	1.4925	1,112
HC8	3633	3678	0.921	3.34	1.012	9435	10571	0.942	1.206	1,120
HC9	2834	3678	0.868	4.16	1.298	9829	10630	0.934	1.137	1,081
HC10	1997	2890	0.79	3.58	1.447	8348	8610	0.963	1.5	1,031
HC11	3950	3876	0.923	3.65	0.981	11221	10899	0.959	0.831	0,971
HC12	5393	4957	0.879	3.68	0.919	10624	11229	0.942	1.6461	1,057
HC13	6124	5269	0.884	3.1	0.860	10411	11048	0.947	1.3645	1,061
HC14	4899	4632	0.931	2.3	0.945	8629	11241	0.77	9.1	1,303
HC15	3354	3430	0.903	0.77	1.023	11416	11239	0.968	1.01	0,984
HC16	3921	3546	0.921	2.75	0.904	11904	11740	0.956	0.86	0,986
HC17	2842	2721	0.896	3.41	0.957	7334	7416	0.941	1.82	1,011
HC18	4415	4712	0.906	3.75	1.067	8822	9411	0.941	2.1	1,067
HC19	3164	3028	0.955	2.98	0.957	11487	11571	0.954	1.264	1,007
HC20	3923	3722	0.935	2.56	0.949	10382	10375	0.953	1.76	0,999
HC21	4720	4849	0.917	3.17	1.027	9943	10185	0.938	1.4906	1,024
HC22	5510	5495	0.936	2.18	0.997	9921	10218	0.945	2.05	1,030
HC23	4379	4642	0.924	3.79	1.060	9239	9313	0.932	1.479	1,008
HC24	3684	3441	0.943	2.206	0.934	10002	9918	0.942	8.2	0,992
HC25	3679	3897	0.911	3.58	1.059	8549	10678	0.855	2.13	1,249
HC26	3900	3897	0.939	4.82	0.999	11528	11684	0.940	2.354	1,014
HC27	4245	4432	0.906	3.14	1.044	10665	11088	0.935	1.266	1,040
HC28	4305	4485	0.875	3.21	1.042	8818	9172	0.954	1.09	1,040
HC29	4709	4617	0.922	3.12	0.980	11251	11716	0.945	1.09	1,041
HC30	3365	3085	0.885	2.2	0.917	11283	11868	0.955	1.699	1,052
***Mean***	**4090.6**	**4031.6**	**0.91**	**3.004**	**0.999**	**9978.83**	**10440.4**	**0.93**	**1.966**	**1.05**
*St.dev*	*893.41*	*753.04*	*0.03*	*0.757*	*0.116*	*1133.31*	*1040.2*	*0.03*	*1.8624*	*0.07*

**Table 2:** LABS performance on healthy controls: second rater.

*Second Rater*	MIDBRAIN					PONS				
Healthy Controls	Manual Volume	LABS Volume	DICE	Hausdorff distance	Volume Ratio	Manual Volume	LABS Volume	DICE	Hausdorff distance	Volume Ratio
HC1	5693	4981	0.907	2.72	0.875	10610	10248	0.941	1.121	0.965
HC2	3733	3380	0.927	3.4	0.905	10541	10341	0.942	1.4	0.981
HC3	4487	3899	0.888	1.02	0.868	10337	10701	0.955	1.226	1.035
HC4	4124	3998	0.919	2.91	0.969	9698	9532	0.958	0.99	0.982
HC5	3280	3153	0.919	2.43	0.961	8907	9284	0.952	1.306	1.042
HC6	5234	4922	0.927	2.21	0.940	10863	11351	0.946	1.4271	1.045
HC7	3710	3636	0.94	2.9	0.980	10043	9935	0.97	0.72	0.989
HC8	3572	3678	0.919	2.87	1.03	9948	10571	0.956	0.86	1.062
HC9	2598	3678	0.818	3.58	1.41	10441	10630	0.952	1.335	1.018
HC10	1978	2890	0.78	3.64	1.461	8541	8610	0.964	0.79	1.008
HC11	3960	3876	0.916	3.607	0.978	11056	10899	0.963	1.24	0.985
HC12	5165	4957	0.89	3.47	0.959	10784	11229	0.961	0.84	1.041
HC13	5940	5269	0.89	3.59	0.887	10705	11048	0.961	0.8481	1.032
HC14	5000	4632	0.927	3.42	0.926	10673	11241	0.8	8.45	1.053
HC15	3427	3430	0.931	1.11	1.0	10919	11239	0.957	1.69	1.029
HC16	3990	3546	0.917	3.1	0.888	11829	11740	0.961	0.75	0.992
HC17	2931	2721	0.887	2.56	0.928	6936	7416	0.948	2.23	1.069
HC18	4564	4712	0.91	3.2	1.032	8752	9411	0.945	1.91	1.075
HC19	3192	3028	0.945	2.4	0.948	11551	11571	0.951	1.11	1.0017
HC20	4012	3722	0.925	2.1	0.927	10234	10375	0.967	5.86	1.013
HC21	4810	4849	0.909	2.89	1.008	10035	10185	0.964	6.74	1.015
HC22	5224	5495	0.88	2.78	1.051	10177	10218	0.96	1.62	1.004
HC23	4472	4642	0.912	3.67	1.038	9252	9313	0.945	0.77	1.006
HC24	3803	3441	0.931	2.21	0.904	9874	9918	0.964	7.65	1.0044
HC25	3664	3897	0.904	3.16	1.063	8469	10678	0.874	1.443	1.26
HC26	3930	3897	0.92	4.21	0.991	11852	11684	0.954	1.44	0.985
HC27	4004	4432	0.9	3.14	1.106	10874	11088	0.959	1.57	1.019
HC28	4419	4485	0.91	3.128	1.014	9177	9172	0.974	1.31	0.999
HC29	4461	4617	0.88	2.72	1.034	11538	11716	0.962	1.03	1.015
HC30	3321	3085	0.91	2.68	0.928	11959	11868	0.975	1.32	0.992
**Mean**	**4089.93**	**4031.6**	**0.9**	**2,895**	**1.0009**	**10219.1**	**10440.4**	**0.95**	**2.033**	**1.024**
*St.dev*	*895.53*	*753.03*	*0.033*	*0,70*	*0.133*	*1144.66*	*1040.2*	*0.033*	*2.112*	*0.052*

**Figure 7:**
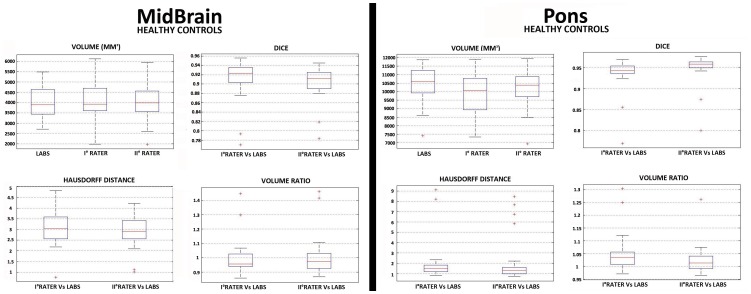
Box and whisker plots of volume quantification, DICE coefficient, Hausdorff Distance and Volume Ratio for each brainstem subregion in healthy controls group who underwent morphological examination using a 3T scanner (Discovery MR-750, GE). Values outside ranges are plotted individually (red cross).

To evaluate the accuracy of the LABS's parcellation compared to the gold standard (represented by manual segmentation) we used several MRI metrics (Table 1 and Table 2; Figure 7). A) DICE coefficient; the mean and standard deviation was excellent: (first/second rater) 0.91±0.03/0.9±0.03 for mibrain; 0.93±0.03/0.95±0.03 for the pons. Similarly, the inter-rater variability for DICE coefficients was consistent among raters (mean ± SD: 0.95±0.02; 0.97±0.03; respectively for the midbrain and pons). B) Volume Ratio; calculation of the volume ratio between the entire midbrain and pons as provided by LABS with respect to the gold standard confirmed the elevated accuracy of our method showing a slight tendency of LABS to overestimate morphology of the pons (mean ± SD volume ratio for the first/second rater: 0.999±0.11/1.0009±0.133 for midbrain; 1.05±0.07/1.024±0.05 for the pons). C) Hausdorff distance; examining the surface distances led to more meaningful comparisons between structures as only accuracy of the segmentation boundaries is taken into account. Our tabulated results showed an increased LABS surface distances over the midbrain (mean ± SD for the first/second rater: 3.005±0.75/2.895±0.7), while the LABS segmentation boundaries followed the manual gold standard boundaries better over the pons (mean ± SD for the first/second rater: 1.966±1.86/2.03±2.01), although a large standard deviation among measurements was detected.

### LABS validation on neurological population

**Figure 8:**
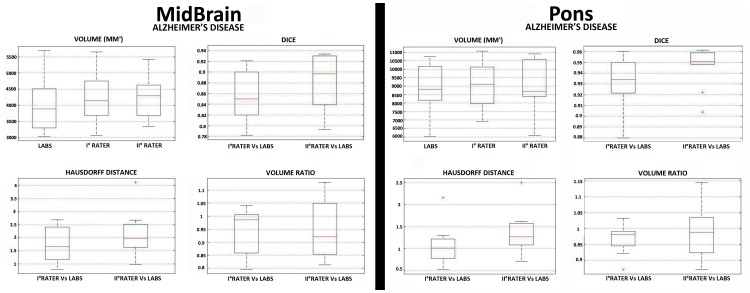
Box and whisker plots of volume quantification, DICE coefficient, Hausdorff Distance and Volume Ratio for each brainstem subregion in a selected population of AD patients (n°10) extracted from ADNI database (Siemens 1.5T, ADNI database). Values outside ranges are plotted individually (red cross).

To further stress the robustness of our MRI-based automated segmentation method, we analysed MRIs of a neurological population. In particular, from the initial cohort we compared the LABS's performance against manual segmentation on randomly selected 10 AD patients extracted from ADNI database (Table S1). Figure S14 showed a qualitative evaluation of the overall 3D reconstruction of the pons and midbrain in the selected AD patients as performed by LABS. Table 3–4 and Figure 8 described quantitative evaluations of LABS's performance by means of several MRI metrics. A) We obtained DICE values ranging from 0.86 (±0.05) to 0.88 (±0.05) for the midbrain, while LABS performed better considering the pons: values ranging from 0.93 (±0.02) to 0.94 (±0.02). Similarly, the inter-rater variability for DICE coefficients was consistent among raters (mean ± SD: 0.91±0.08; 0.95±0.06; respectively for the midbrain and pons). B) Calculation of the volume ratio between the entire midbrain and pons as provided by LABS with respect to the gold standard confirmed the elevated accuracy of our method also considering neurological patients, with values around 0.95 for the midbrain and values around 0.98 for the pons (mean ± SD volume ratio for the first/second rater: 0.95±0.09/0.95±0.11 for midbrain; 0.97±0.05/0.98±0.08 for the pons). C) Hausdorff distance; similar to that found in healthy controls, tabulated results showing an increased LABS surface distances over the midbrain (mean ± SD for the first/second rater: 1.71±0.68/2.15±0.83), while the LABS segmentation boundaries followed the manual gold standard boundaries better over the pons (mean ± SD for the first/second rater: 1.07±0.46/1.33±0.59).

**Table 3:** LABS performance on patients with Alzheimer's Disease: first rater.

*First Rater*	MIDBRAIN					PONS				
AD_ID	Manual Volume	LABS Volume	DICE	Hausdorff distance	Volume Ratio	Manual Volume	LABS Volume	DICE	Hausdorff distance	Volume Ratio
012_S_0689	5655	5688	0,839	2,685	1,006	10127	10171	0,949	0,691	1,004
033_S_0733	4750	3938	0,782	2,442	0,829	10733	10618	0,95	0,530	0,989
136_S_0300	5393	5420	0,9	1,2	1,005	9343	8838	0,929	2,160	0,945
027_S_1081	3072	3044	0,82	1,72	0,991	8855	8168	0,914	1,060	0,922
032_S_1101	4402	4509	0,92	1,168	1,024	7967	8220	0,88	1,221	1,031
033_S_0724	3840	3300	0,860	2,411	0,859	7079	6890	0,938	0,785	0,973
131_S_0457	3371	3306	0,82	0,801	0,981	8862	8785	0,96	0,822	0,991
127_S_0844	3689	3839	0,873	1,628	1,041	6902	6013	0,921	1,303	0,871
133_S_1170	3880	3092	0,822	2,1	0,797	9716	9608	0,924	1,177	0,988
136_S_0426	4577	4305	0,921	0,91	0,941	11065	10745	0,96	0,971	0,971
***Mean***	**4262,9**	**4044,1**	**0,86**	**1,71**	**0,95**	**9064,9**	**8805,6**	**0,93**	**1,07**	**0,97**
*St.dev*	*845,83*	*939,16*	*0,05*	*0,68*	*0,09*	*1426,92*	*1555,4*	*0,02*	*0,46*	*0,05*

**Table 4:** LABS performance on patients with Alzheimer's Disease: second rater.

*Second Rater*	MIDBRAIN					PONS				
AD_ID	Manual Volume	LABS Volume	DICE	Hausdorff distance	Volume Ratio	Manual Volume	LABS Volume	DICE	Hausdorff distance	Volume Ratio
012_S_0689	5420	5688	0,839	4,1193	1,0494	10826	10171	0,952	1,337	0,939
033_S_0733	4609	3938	0,793	2,67	0,854	10567	10618	0,958	1,1847	1,004
136_S_0300	4800	5420	0,934	0,9821	1,129	8394	8838	0,959	2,4965	1,052
027_S_1081	3342	3044	0,897	1,98	0,91	9371	8168	0,904	1,578	0,871
032_S_1101	4585	4509	0,93	1,628	0,983	8900	8220	0,950	1,264	0,923
033_S_0724	4018	3300	0,866	1,9878	0,821	7544	6890	0,948	0,7166	0,913
131_S_0457	3678	3306	0,897	1,6393	0,898	8484	8785	0,960	0,7282	1,035
127_S_0844	3541	3839	0,93	2,5216	1,084	6076	6013	0,961	1,2825	0,989
133_S_1170	3800	3092	0,833	2,12	0,813	8398	9608	0,922	1,088	1,144
136_S_0426	4623	4305	0,919	1,8703	0,931	10904	10745	0,95	1,618	0,985
***Mean***	**4241,6**	**4044,1**	**0,884**	**2,152**	**0,948**	**8946,4**	**8805,6**	**0,946**	**1,329**	**0,986**
*St.dev*	*663,45*	*939,16*	*0,049*	*0,83*	*0,11*	*1534,76*	*1555,39*	*0,018*	*0,509*	*0,079*

Since previous post-mortem and neuroimaging studies demonstrated that patients with AD might be characterized by early abnormalities of these two brainstem subregions [20], [21], we further evaluated the presence of morphological atrophy, comparing the brainstem volumes, as assessed by LABS, on a larger sample size of AD patients and healthy controls (n° 40 for each group). Unpaired t-test revealed significant findings when comparing the pons and midbrain volumes between groups. The pons volume in AD patients resulted to be strongly reduced (∼10%) with respect to controls (T_-value_ = 4.48, p-level = 0.00002; mean ± SD: 10693.02±917.2 mm^3^, 9587.266±1260.4 mm^3^; respectively for controls and AD), whereas the statistical difference detected in the midbrain was less prominent (8.2%)(T_-value_ = 2.89, p-level = 0.004; mean ± SD: 4864.21±492.3 mm^3^, 4461.6±729.8 mm^3^; respectively for controls and AD)(Table S1 and Table S2). To further confirm this later evidence we performed a voxel-based analysis on the same dataset using a well-known unbiased advanced neuroimaging tool: voxel-based morphometry (VBM). The objective quantification of volumetric loss in the brainstem subregions, as performed by LABS, was also confirmed by this different MRI approach (see Figure S15).

## Discussion

Segmenting sub-cortical structures from 3D brain images has attracted much attention in recent years given the numerous clinical and neuroscientific applications. Until now, a large amount of advanced post-processing MRI tools have been developed allowing very reliable and consistent objective quantification of almost all subcortical regions of the human brain. However, two clinically relevant regions, such as the pons and midbrain, have received very little attention. As recently stated by Lambert et al., [6], this is in part due to the difficulties in resolving *in vivo* the internal architecture of the brainstem in a reliable and repeatable fashion. We present here a novel MRI method, called LABS, to automatically segment these two complex brain regions.

Overall, LABS may be considered a part of the knowledge-driven methods [14]–[16] that generally use implicit or explicit anatomical knowledge to guide the segmentation, performing better when applied on single brain structures. Generally, this class of automatic 3D segmentation methods presents difficulties if pathology, scan sequence or manual delineation protocol differs from those that the method is designed for [14]. Our data did not support this hypothesis. Indeed, the robustness and accuracy of our method was tested by means of quantitative evaluations both on a large group of healthy subjects and on neurological patients characterized by evident anatomical abnormalities in the brainstem [20], [21]. Moreover, further evaluation of our method has also been reached including MRIs acquired with a different scanner and protocol (ADNI database) [25]. On the entire set of healthy controls, quantitative comparison of the LABS's segmentations against the corresponding manual measurements showed excellent performance considering all reliability metrics. In the neurological realm, our software showed high performance in the segmentation of the pons, while more variability was detected in the quantification of the midbrain volume. However, when we directly compared the volumetric measurements of the brainstem subregions, as obtained from LABS, between controls and AD patients we found significant atrophies of the pons and midbrain. Overall, it is important to highlight that among all MRI metrics, LABS's performance had slightly lower accuracy when distance-based measurements were considered. In fact, the Hausdorff distance value, a measure strongly influenced by the shape and surface of the brain region, was higher in the midbrain with respect to pons, thus confirming the intrinsic anatomical complexity of this subcortical region.

To the best of our knowledge, LABS is the first advanced neuroimaging technique able to automatically segment the pons and midbrain of the human brain. State-of-the-art methods for human brain MRI segmentation using conventional (manual morphometry) [19], [22], [23], [26] and advanced automatic methods [3]–[5], [7]–[16] are not applicable to clearly separate the midbrain and pons. LABS represents the first approach that clearly distinguishes these two brainstem subregions without requiring manual intervention. In fact, the segmentation and labeling of the pons and midbrain are performed using a revised landmark-based approach that integrates the well-known thresholding-based approach, with the individuation of specific anatomical markers (i.e., corpus callosum, mamillary body and quadrigeminal plate) useful to better identify and separate the brainstem subregions. Generally, automated segmentation methods, using landmark-based approach, have a more widespread applicability, mainly in neurological context [8]. However, in contrast to atlas or template-based methods (where atlas definition is highly dependent upon the population that they were obtained from), LABS relies heavily on anatomical landmarks present in all neurological and psychiatric populations.

Despite the excellent performance provided by LABS, our method has some limitations that deserve to be discussed. First, the robustness of our method relies heavily on the intensity thresholding of MRIs. Generally, intensity-based classification of MRIs has proven problematic. Intra-scan intensity inhomogeneities due to radio-frequency coils or acquisition sequences are a common source of difficulty that ultimately may disturb intensity-based segmentation methods. Although we also demonstrated the robustness of our method on a different scanner and protocol (ADNI database), a future target should be the inclusion of additional algorithms able to adapt intra-scan and inter-scan intensity inhomogeneities, by using *a priori* knowledge of tissue proprieties and voxel-intensity [5]. Another limitation was the chosen anatomical boundaries for the separation of the pons and midbrain from surrounding structures. In particular, as regards the separation of the middle cerebellar peduncles from the pons, due to the lack of shared anatomical criteria, we only referred to one previous morphometric criterium [19] to extract useful landmarks. However, to improve the separation between the pons and middle cerebellar peduncles, we retain that an useful future improvement will derive from the implementation of a multimodal approach, integrating diffusion tensor images (DTI) information in the segmentation steps of our algorithm. With DTI, it would be possible to deeply delineate the boundary between the brainstem and the cerebellar peduncles using cerebellar tracts to place new reliable anatomical landmarks, Final, the computation of the midsagittal plane was restricted to planes parallel to the orientation of the volume after registration, without sub-voxel accuracy – as opposed to other midsagittal plane detection algorithms [43]. Although it has been proposed that this does not seem to be of critical importance in 3D brain images [12], we retain that this lack of precision had a negligible impact on our method since segmentation steps specifically relied on well-defined anatomical landmarks.

In conclusion, after additional developments (i.e., automatic adaptive correction of intensity inhomogeneities for segmenting MRIs), we retain that our method might represent a useful tool for future applications in clinical practice.

## Supporting Information

Figure S1 **Gravity center of the headmask obtained using Otzu's binarization.**
(DOCX)

Figure S2 **Automatic segmentation of the corpus callosum on different slices included in the subvolume S^1^ within the same subject.**
(DOCX)

Figure S3 **Automatic segmentation of the upper part of the brainstem (included in the R^1^ region) in different slices within the same subject.**
(DOCX)

Figure S4 **The mid-sagittal slice can be detected as the slice where is maximal the expansion of Sylvius aqueduct in accordance with morphological knowledge.**
(DOCX)

Figure S5 **Area of the upper part of the brainstem in each single slice within the subvolume S^1^, as calculated in one single subject.** The point of minimum corresponds to the position of mid-sagittal slice.
(DOCX)

Figure S6 **Contours of brainstem.** The red cross represents the point used to separate the rostral midbrain from the mamillary body.
(DOCX)

Figure S7 **Segmentation of quadrigeminal plate using the R^1^ region (A), deleting the pixels belonging to the brainstem (B) and identifying the plate as the connected component that had the center of gravity closer to the midbrain tectum (C).**
(DOCX)

Figure S8 **Separation of cerebellum from posterior brainstem (right side) using a coronal plane through two points belonging to the dorsum of the brainstem (left side).**
(DOCX)

Figure S9 **Automatic 2D segmentation of midbrain.**
(DOCX)

Figure S10 **Automatic 2D segmentation of pons.**
(DOCX)

Figure S11 **Arbitrary anatomical landmark useful for separating cerebellar peduncles from pons.** Figure displayed a volumetric slab of 40 mm (0.5-mm section thickness) tangent to the floor of the fourth ventricle (left side), placed on a mid-sagittal plane to cover the entire extension of the superior cerebellar peduncles (right side).
(DOCX)

Figure S12 **Individuation of two bilateral points employed as anatomical landmarks of the middle cerebellar peduncles.**
(DOCX)

Figure S13 **Separation of middle cerebellar peduncle from pons using two vertical lines.**
(DOCX)

Figure S14 **3D segmentation of pons and midbrain as performed by LABS for each AD patient.**
(DOCX)

Figure S15 **VBM analysis revealed the presence of significant volumetric WM loss in the midbrain and pons of AD patients compared to healthy controls.** In order to further validate measurement of brainstem as performed by LABS, we employed voxel-based morphometry (VBM) in order to reveal subtle volumetric loss in AD patients. Data were processed using the SPM8 software where we applied VBM implemented in the VBM8 toolbox, incorporating the DARTEL toolbox that was used to obtain a high-dimensional normalization protocol (http://dbm.neuro.uni-jena.de/vbm.html). Images were bias-corrected, tissue classified, and registered using linear (12-parameter affine) and non-linear transformations, within a unified model. Subsequently, the warped white matter (WM) segment was affine transformed into MNI space and were scaled by the Jacobian determinants of the deformations (modulation). Finally, the modulated volumes were smoothed with a Gaussian kernel of 8 mm. The WM volume maps were statistically analysed using the general linear model based on Gaussian random field theory. We investigated the presence of volumetric differences between AD patients (n°40) and healthy controls (n°40) using unpaired *t*-test. Age and total intracranial volume (ICV) were included in the model as covariates of no-interest. We selected midbrain and pons as regions of interest (ROIs) for VBM analysis. These ROIs were created with the “aal.02” atlas included in the Wake Forest University Pickatlas software version 1.04 (http://www.fmri.wfubmc.edu/download.htm). Statistical threshold was set at P<0.05 with Family-Wise error (FWE) correction for multiple comparisons within ROIs. As showed in Figure S15, we detected abnormal volumetric losses of the midbrain (P_FWE_ = 0.03; T-value; 3.45; x: 19; y:−18; z:−19) and pons (P_FWE_ = 0.01; T-value; 4.02; x: 10; y:−24; z:−46) in AD patients when compared to age-/sex-matched healthy controls.
(DOCX)

Table S1 **List of AD patients from the ADNI database that were included in the study.**
(DOC)

Table S2 **List of healthy controls from the ADNI database that were included in the study.**
(DOC)
