# Supplementary figures and images for: Fully Automated Segmentation of the Pons and Midbrain Using Human T1 MR Brain Images
Source: PLoS One. 2014 Jan 28;9(1):e85618. doi: 10.1371/journal.pone.0085618 (PMC3904850; doi:10.1371/journal.pone.0085618)

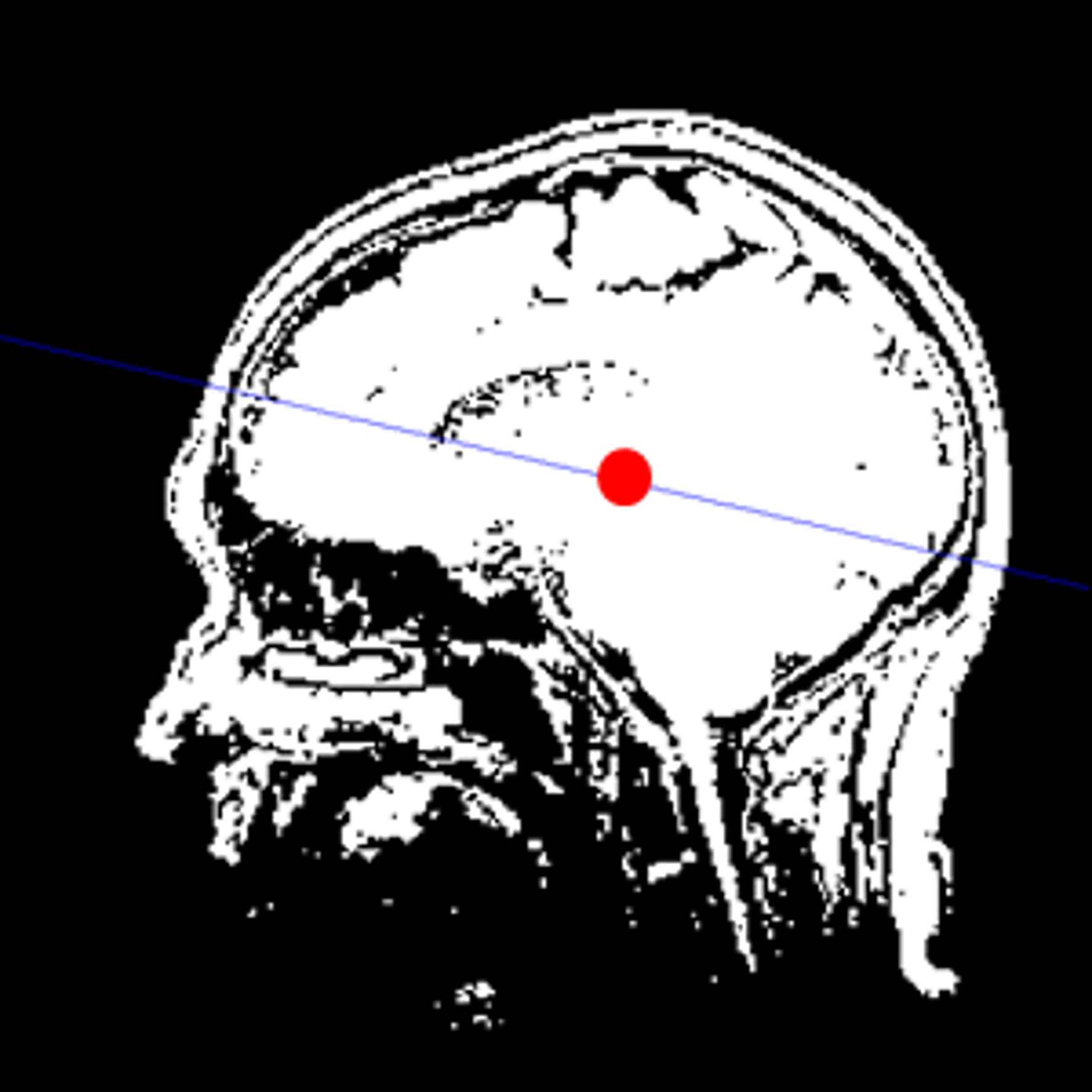


Supplementary Figure 1: Gravity center of the headmask obtained using Otzu’s binarization.

Supplement: Figure S1 — Gravity center of the headmask obtained using Otzu's binarization. (DOCX) [file pone.0085618.s001.docx]

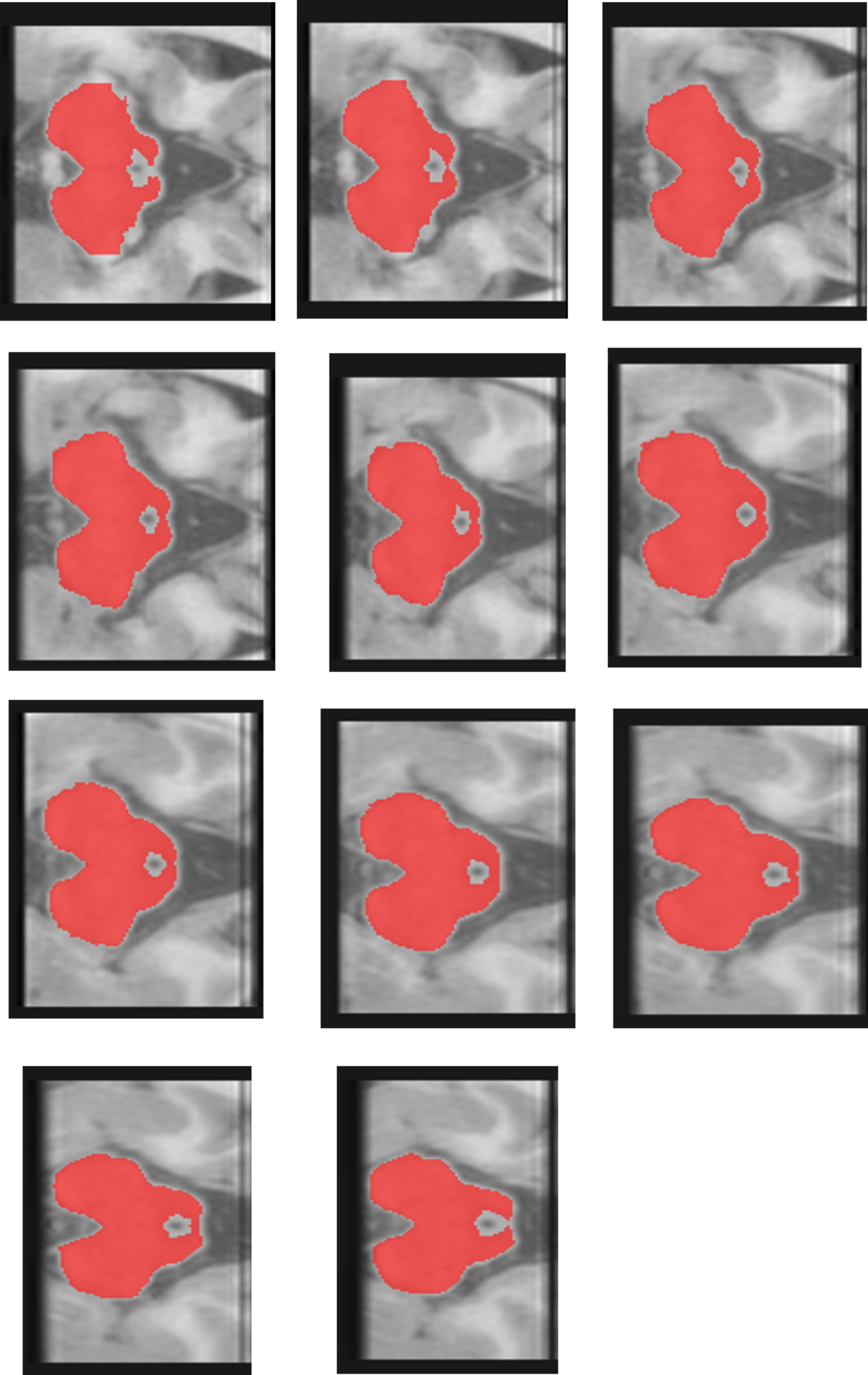


Figure S9: Automatic 2D segmentation of midbrain.

Supplement: Figure S9 — Automatic 2D segmentation of midbrain. (DOCX) [file pone.0085618.s009.docx]

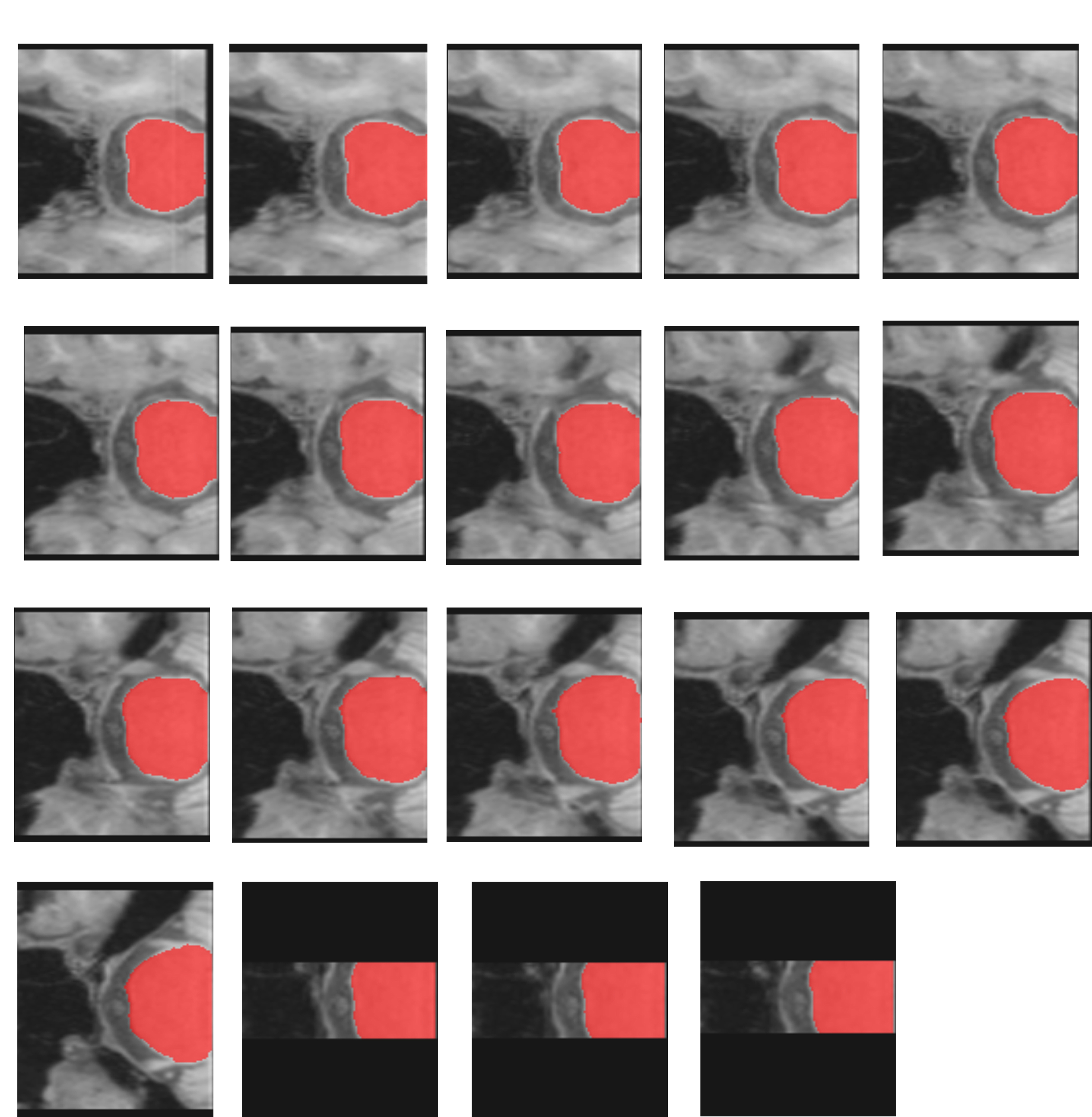


**Figure S10:** Automatic 2D segmentation of pons.

Supplement: Figure S10 — Automatic 2D segmentation of pons. (DOCX) [file pone.0085618.s010.docx]

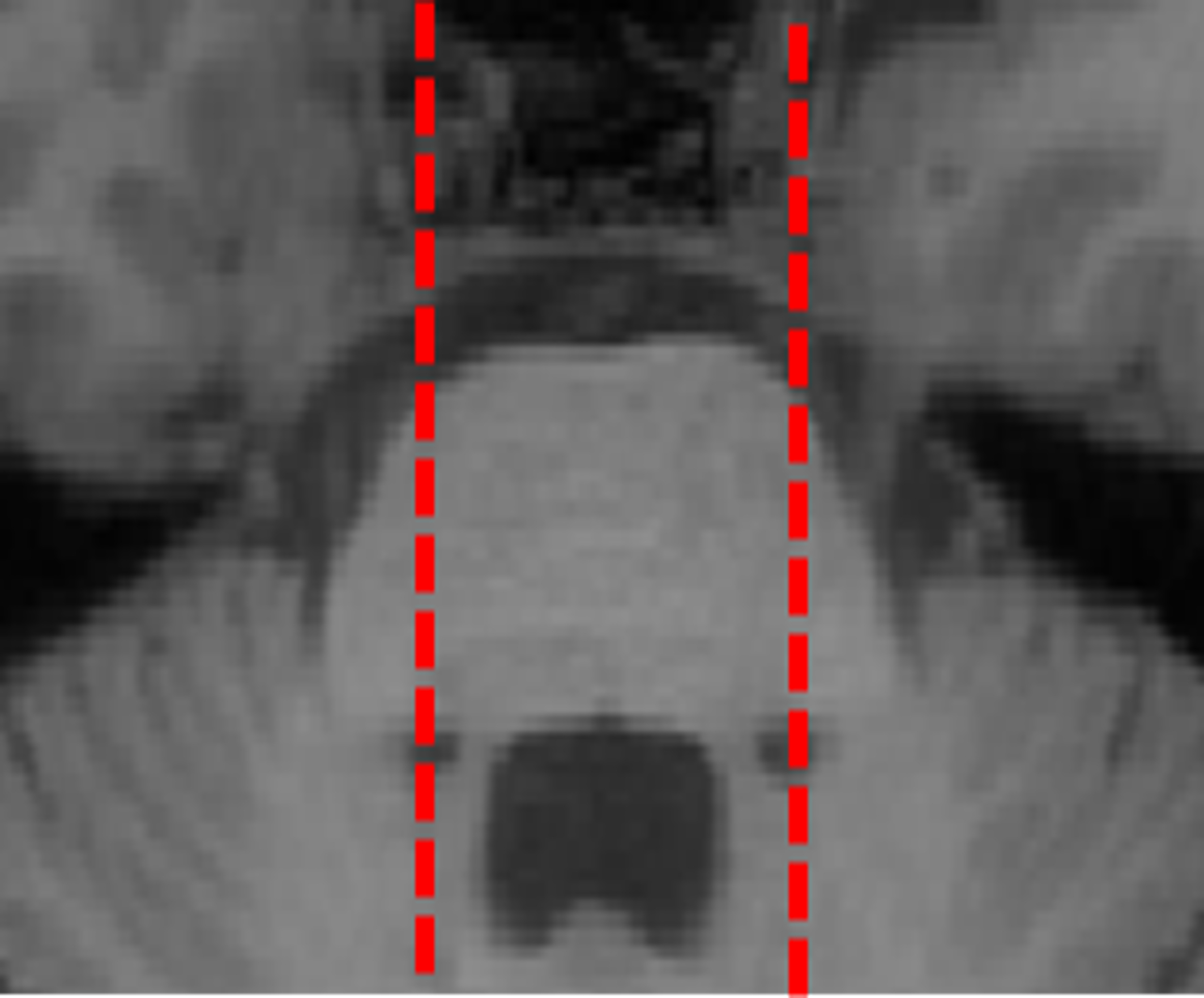


**Figure S13:** Separation of middle cerebellar peduncle from pons using two vertical lines

Supplement: Figure S13 — Separation of middle cerebellar peduncle from pons using two vertical lines. (DOCX) [file pone.0085618.s013.docx]

**
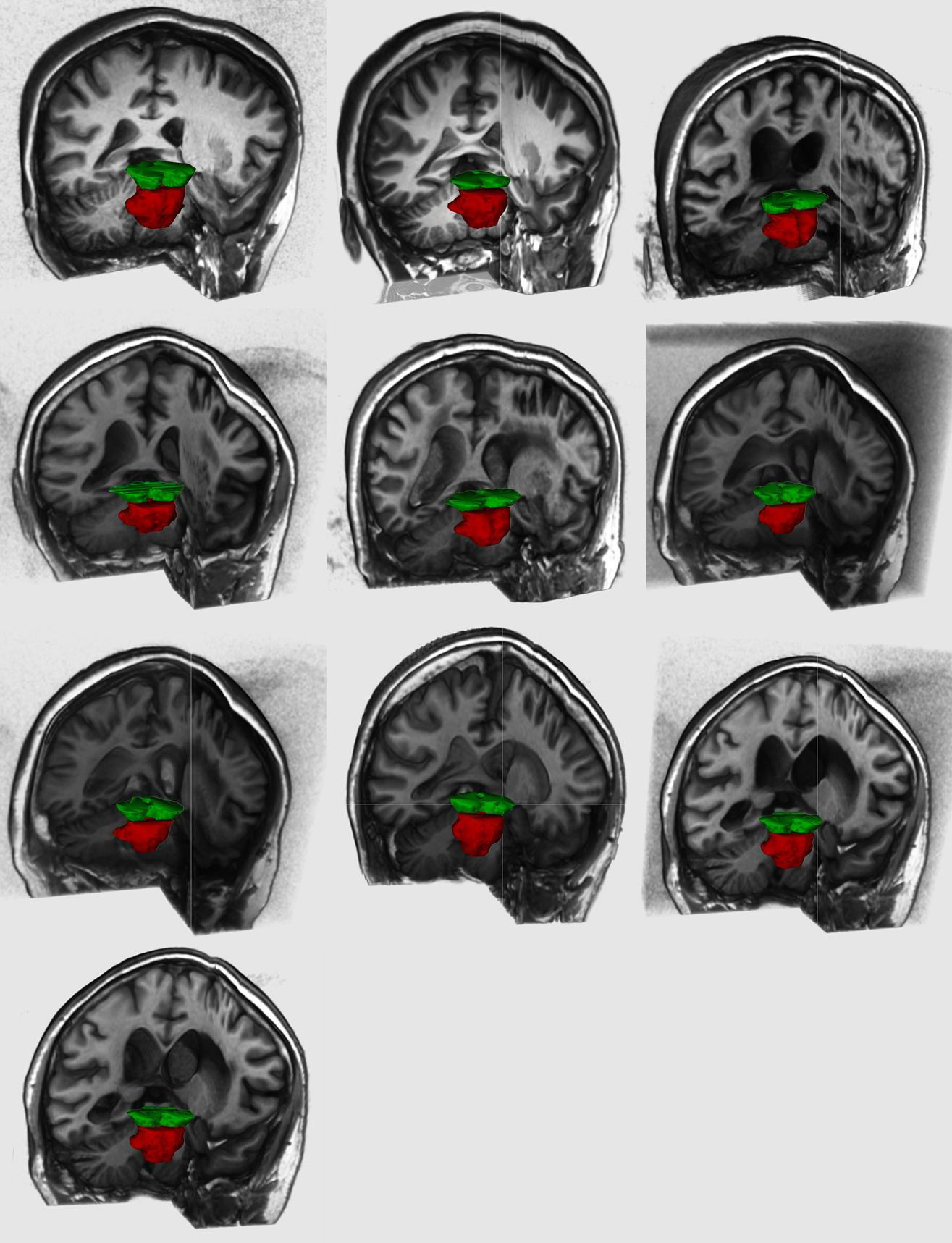
**

**Figure S14**: 3D segmentation of pons and midbrain as performed by LABS for each AD patient.

Supplement: Figure S14 — 3D segmentation of pons and midbrain as performed by LABS for each AD patient. (DOCX) [file pone.0085618.s014.docx]
