# Supplementary material for: Fully Automated Segmentation of the Pons and Midbrain Using Human T1 MR Brain Images
Source: PLoS One. 2014 Jan 28;9(1):e85618. doi: 10.1371/journal.pone.0085618 (PMC3904850; doi:10.1371/journal.pone.0085618)
Supplement: Figure S2 — Automatic segmentation of the corpus callosum on different slices included in the subvolume S1 within the same subject. (DOCX) [file pone.0085618.s002.docx]

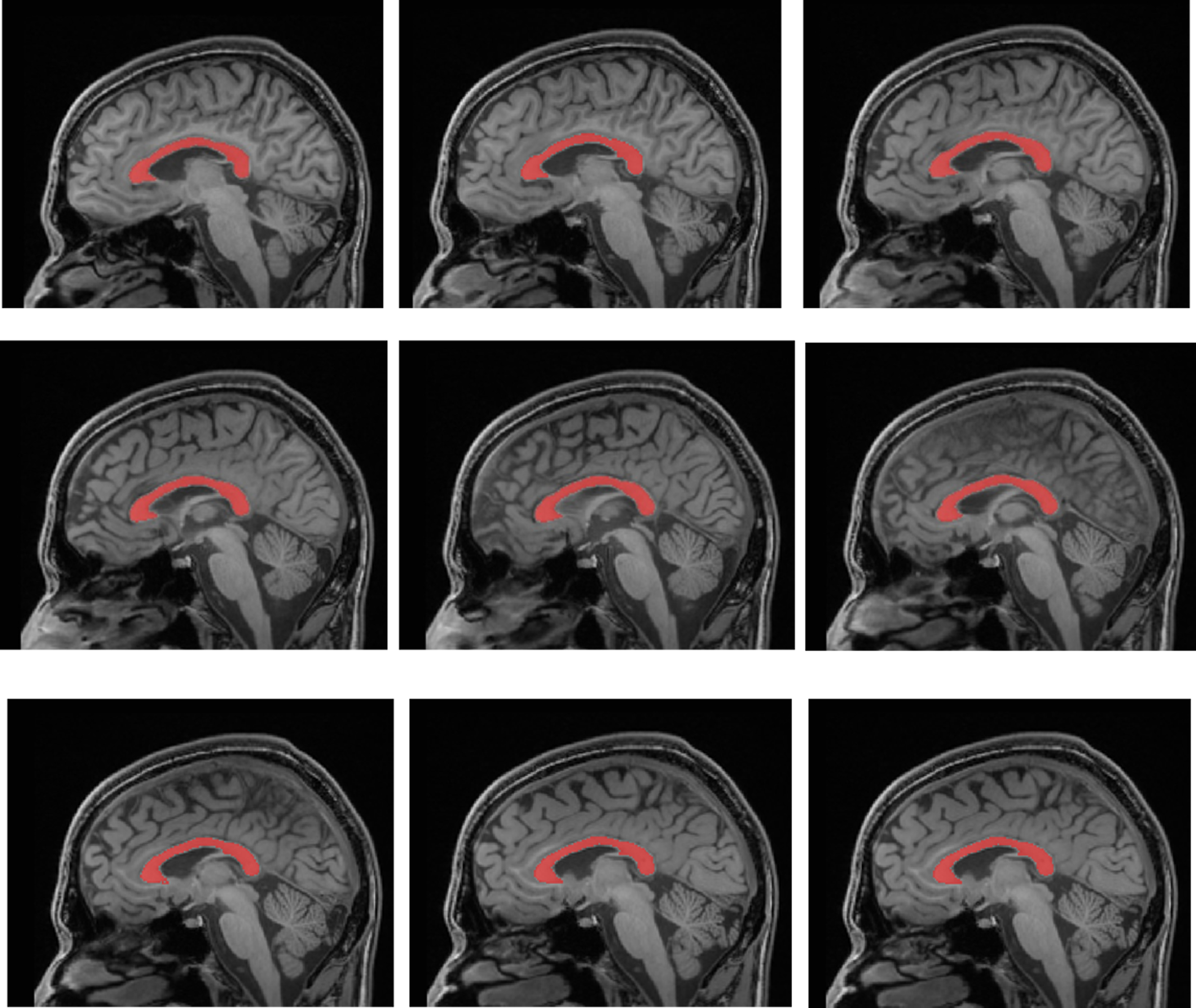


Figure S2: Automatic segmentation of the corpus callosum on different slices included in the subvolume S^1^ within the same subject.
