# Supplementary material for: Fully Automated Segmentation of the Pons and Midbrain Using Human T1 MR Brain Images
Source: PLoS One. 2014 Jan 28;9(1):e85618. doi: 10.1371/journal.pone.0085618 (PMC3904850; doi:10.1371/journal.pone.0085618)
Supplement: Figure S3 — Automatic segmentation of the upper part of the brainstem (included in the R1 region) in different slices within the same subject. (DOCX) [file pone.0085618.s003.docx]

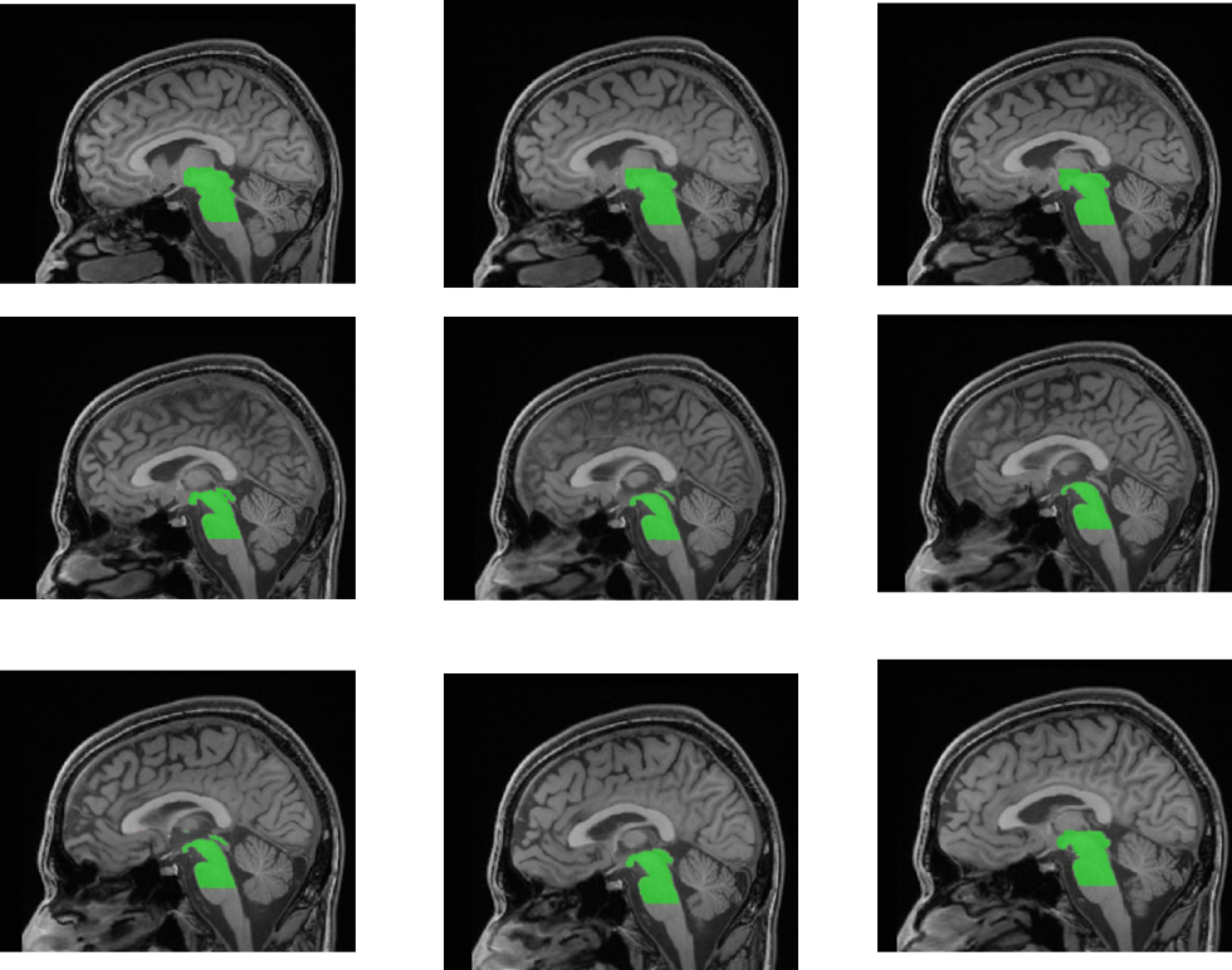


Figure S3: Automatic segmentation of the upper part of the brainstem (included in the R^1^ region) in different slices within the same subject.
