# Supplementary material for: Fully Automated Segmentation of the Pons and Midbrain Using Human T1 MR Brain Images
Source: PLoS One. 2014 Jan 28;9(1):e85618. doi: 10.1371/journal.pone.0085618 (PMC3904850; doi:10.1371/journal.pone.0085618)
Supplement: Figure S4 — The mid-sagittal slice can be detected as the slice where is maximal the expansion of Sylvius aqueduct in accordance with morphological knowledge. (DOCX) [file pone.0085618.s004.docx]

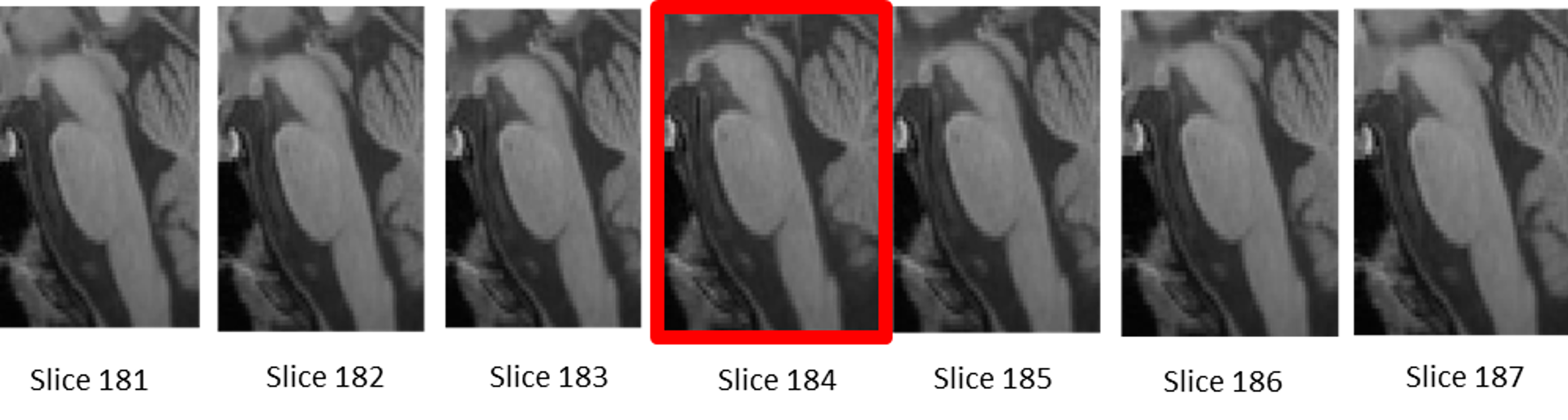


Figure S4: The mid-sagittal slice can be detected as the slice where is maximal the expansion of Sylvius aqueduct in accordance with morphological knowledge.
