# Supplementary material for: Fully Automated Segmentation of the Pons and Midbrain Using Human T1 MR Brain Images
Source: PLoS One. 2014 Jan 28;9(1):e85618. doi: 10.1371/journal.pone.0085618 (PMC3904850; doi:10.1371/journal.pone.0085618)
Supplement: Figure S5 — Area of the upper part of the brainstem in each single slice within the subvolume S1, as calculated in one single subject. The point of minimum corresponds to the position of mid-sagittal slice. (DOCX) [file pone.0085618.s005.docx]

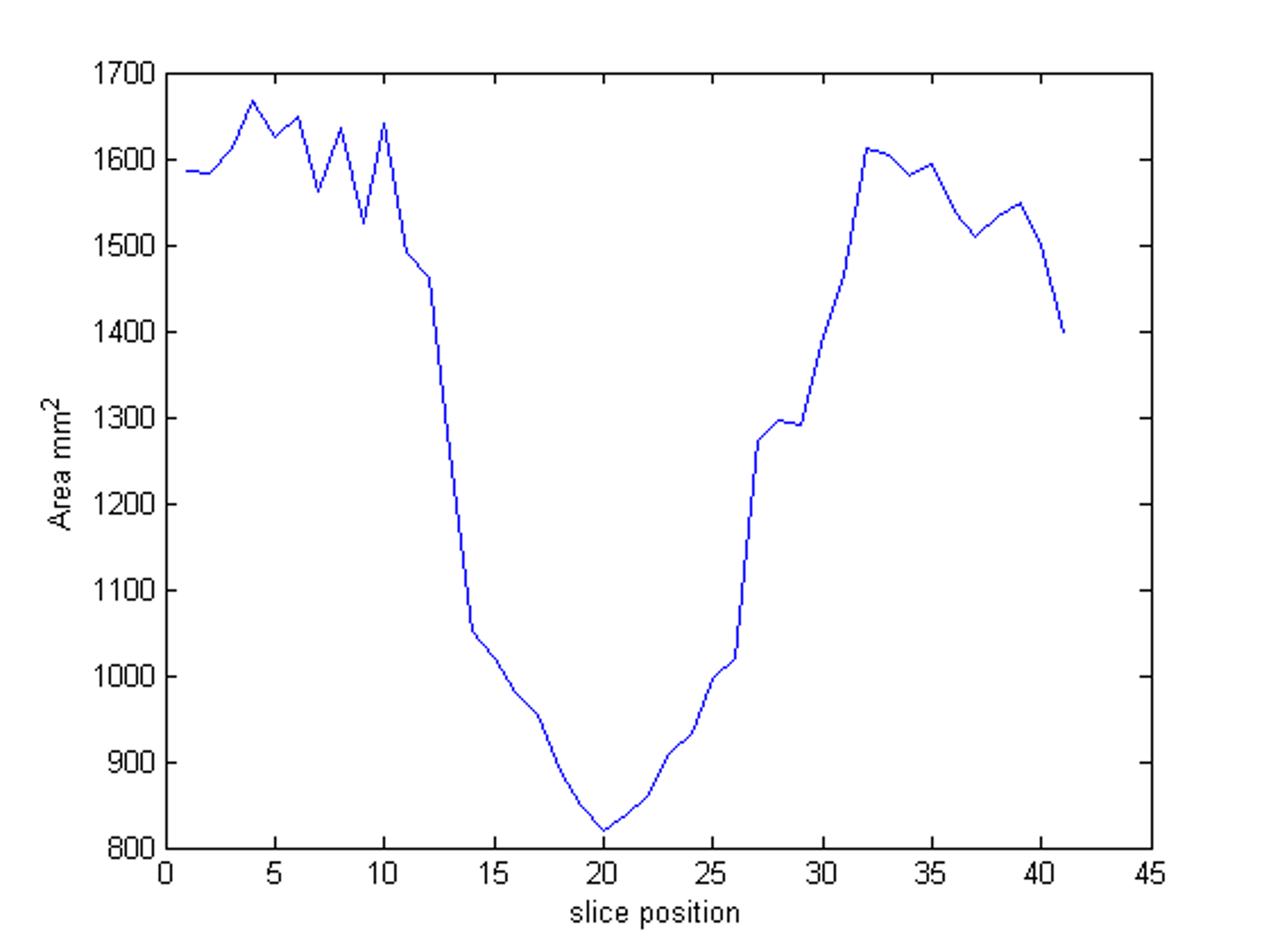


Figure S5: Area of the upper part of the brainstem in each single slice within the subvolume S^1^ , as calculated in one single subject. The point of minimum corresponds to the position of mid-sagittal slice.
