# Supplementary material for: Fully Automated Segmentation of the Pons and Midbrain Using Human T1 MR Brain Images
Source: PLoS One. 2014 Jan 28;9(1):e85618. doi: 10.1371/journal.pone.0085618 (PMC3904850; doi:10.1371/journal.pone.0085618)
Supplement: Figure S6 — Contours of brainstem. The red cross represents the point used to separate the rostral midbrain from the mamillary body. (DOCX) [file pone.0085618.s006.docx]

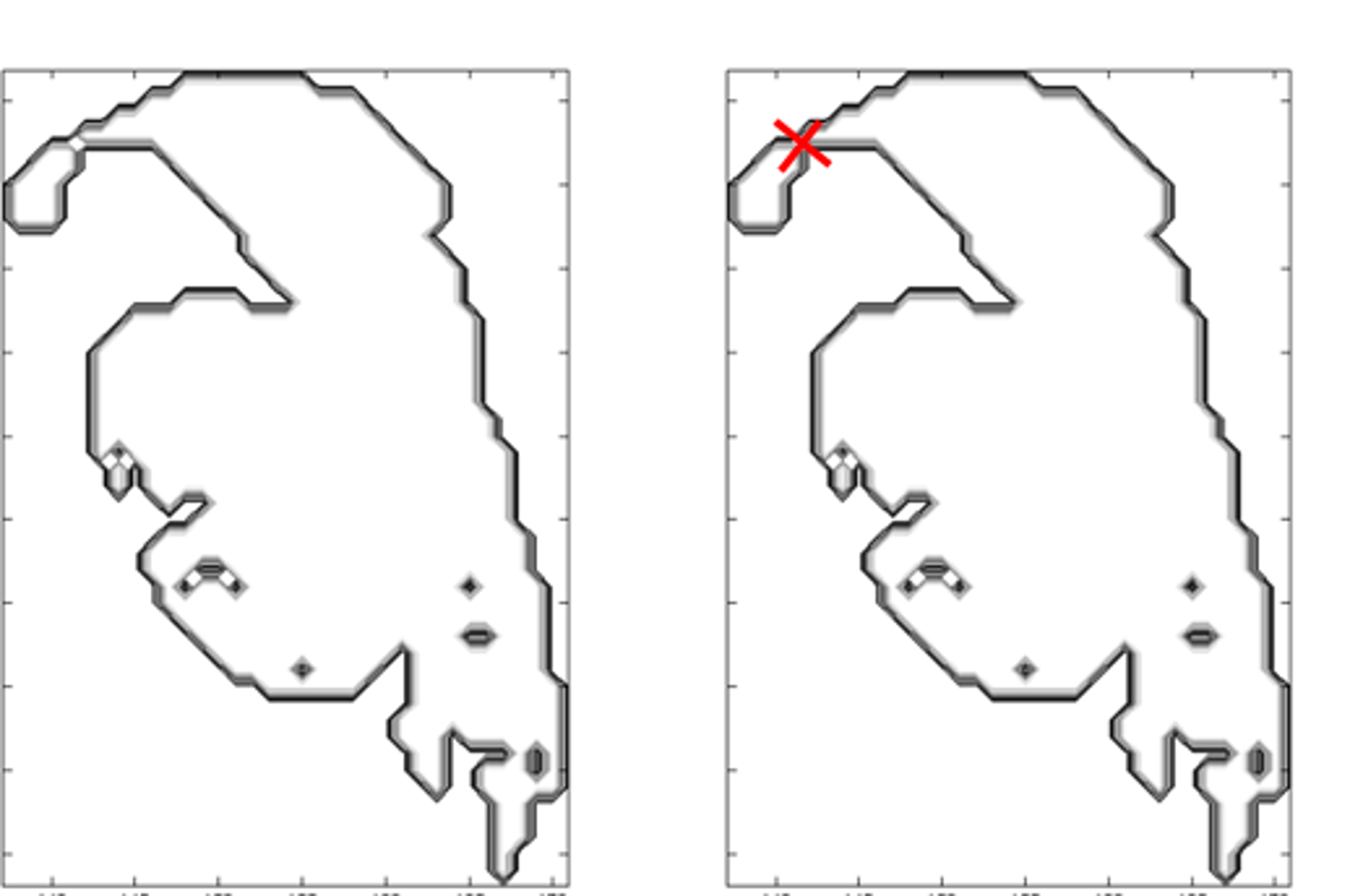


Figure S6: Contours of brainstem. The red cross represents the point used to separate the rostral midbrain from the mamillary body.
