# Supplementary material for: Fully Automated Segmentation of the Pons and Midbrain Using Human T1 MR Brain Images
Source: PLoS One. 2014 Jan 28;9(1):e85618. doi: 10.1371/journal.pone.0085618 (PMC3904850; doi:10.1371/journal.pone.0085618)
Supplement: Figure S7 — Segmentation of quadrigeminal plate using the R1 region (A), deleting the pixels belonging to the brainstem (B) and identifying the plate as the connected component that had the center of gravity closer to the midbrain tectum (C). (DOCX) [file pone.0085618.s007.docx]

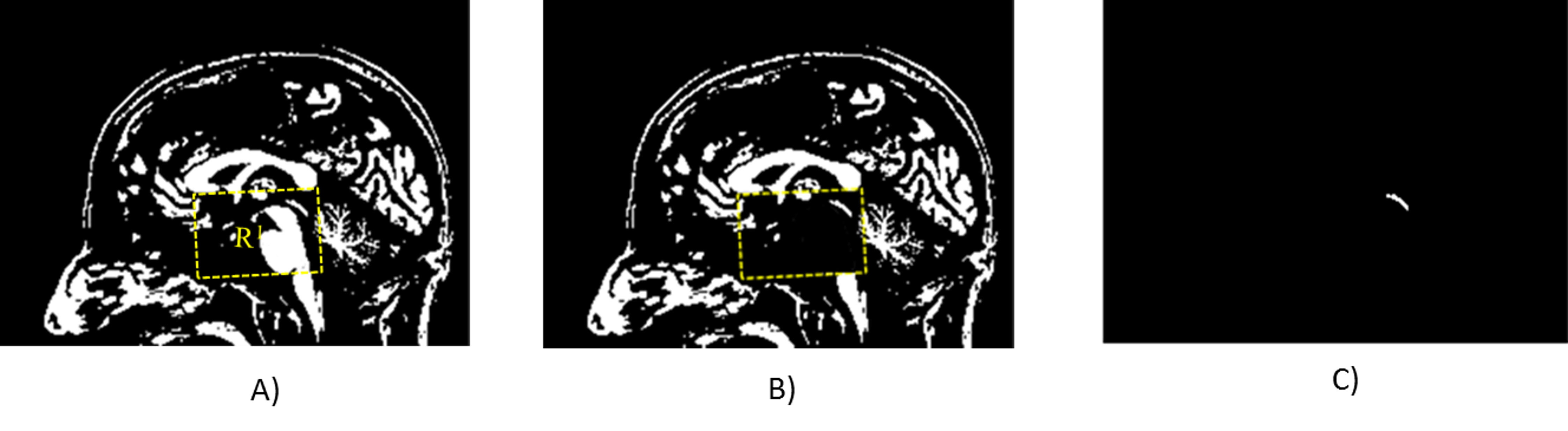


Figure S7: Segmentation of quadrigeminal plate using the R^1^ region (A), deleting the pixels belonging to the brainstem (B) and identifying the plate as the connected component that had the center of gravity closer to the midbrain tectum (C).

ù
