# Supplementary material for: Fully Automated Segmentation of the Pons and Midbrain Using Human T1 MR Brain Images
Source: PLoS One. 2014 Jan 28;9(1):e85618. doi: 10.1371/journal.pone.0085618 (PMC3904850; doi:10.1371/journal.pone.0085618)
Supplement: Figure S8 — Separation of cerebellum from posterior brainstem (right side) using a coronal plane through two points belonging to the dorsum of the brainstem (left side). (DOCX) [file pone.0085618.s008.docx]

**
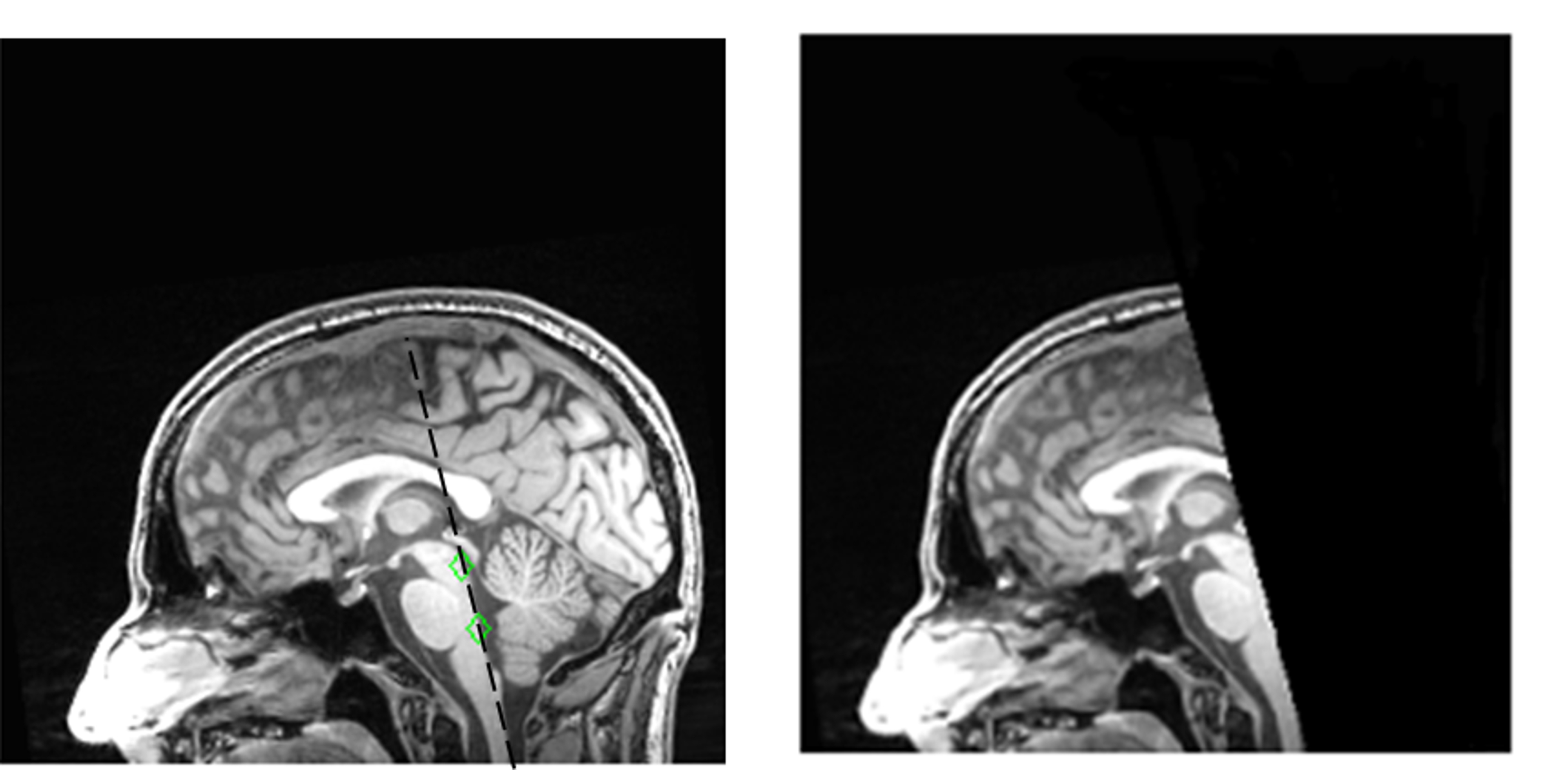
**

**Figure S8:** Separation of cerebellum from posterior brainstem (right side) using a coronal plane through two points belonging to the dorsum of the brainstem (left side).
