# Supplementary material for: Fully Automated Segmentation of the Pons and Midbrain Using Human T1 MR Brain Images
Source: PLoS One. 2014 Jan 28;9(1):e85618. doi: 10.1371/journal.pone.0085618 (PMC3904850; doi:10.1371/journal.pone.0085618)
Supplement: Figure S11 — Arbitrary anatomical landmark useful for separating cerebellar peduncles from pons. Figure displayed a volumetric slab of 40 mm (0.5-mm section thickness) tangent to the floor of the fourth ventricle (left side), placed on a mid-sagittal plane to cover the entire extension of the superior cerebellar peduncles (right side). (DOCX) [file pone.0085618.s011.docx]

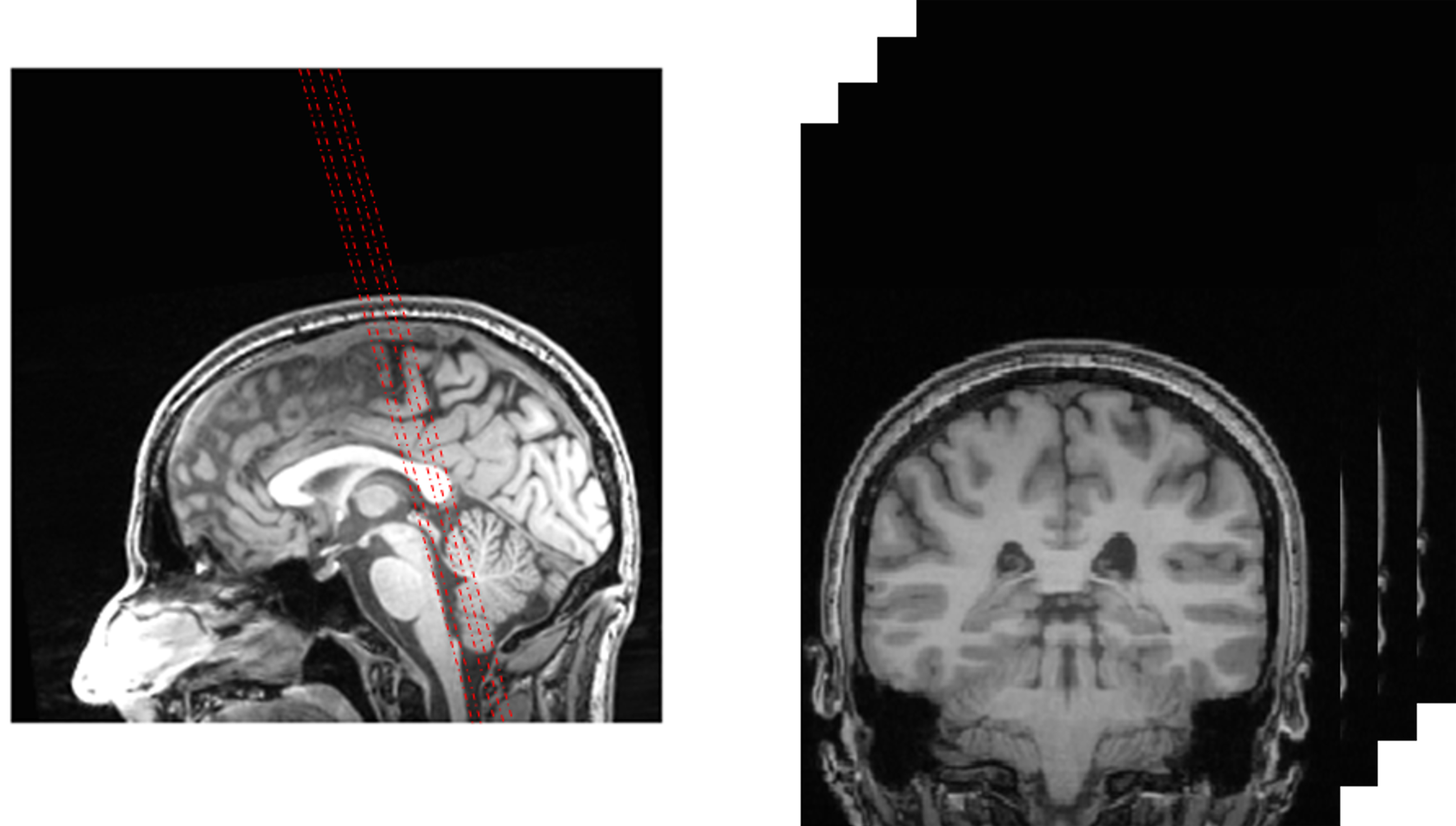


Figure S11: Arbitrary anatomical landmark useful for separating cerebellar peduncles from pons. Figure displayed a volumetric slab of 40 mm (0.5-mm section thickness) tangent to the floor of the fourth ventricle (left side), placed on a mid-sagittal plane to cover the entire extension of the superior cerebellar peduncles (right side).
