# Supplementary material for: Fully Automated Segmentation of the Pons and Midbrain Using Human T1 MR Brain Images
Source: PLoS One. 2014 Jan 28;9(1):e85618. doi: 10.1371/journal.pone.0085618 (PMC3904850; doi:10.1371/journal.pone.0085618)
Supplement: Figure S12 — Individuation of two bilateral points employed as anatomical landmarks of the middle cerebellar peduncles. (DOCX) [file pone.0085618.s012.docx]

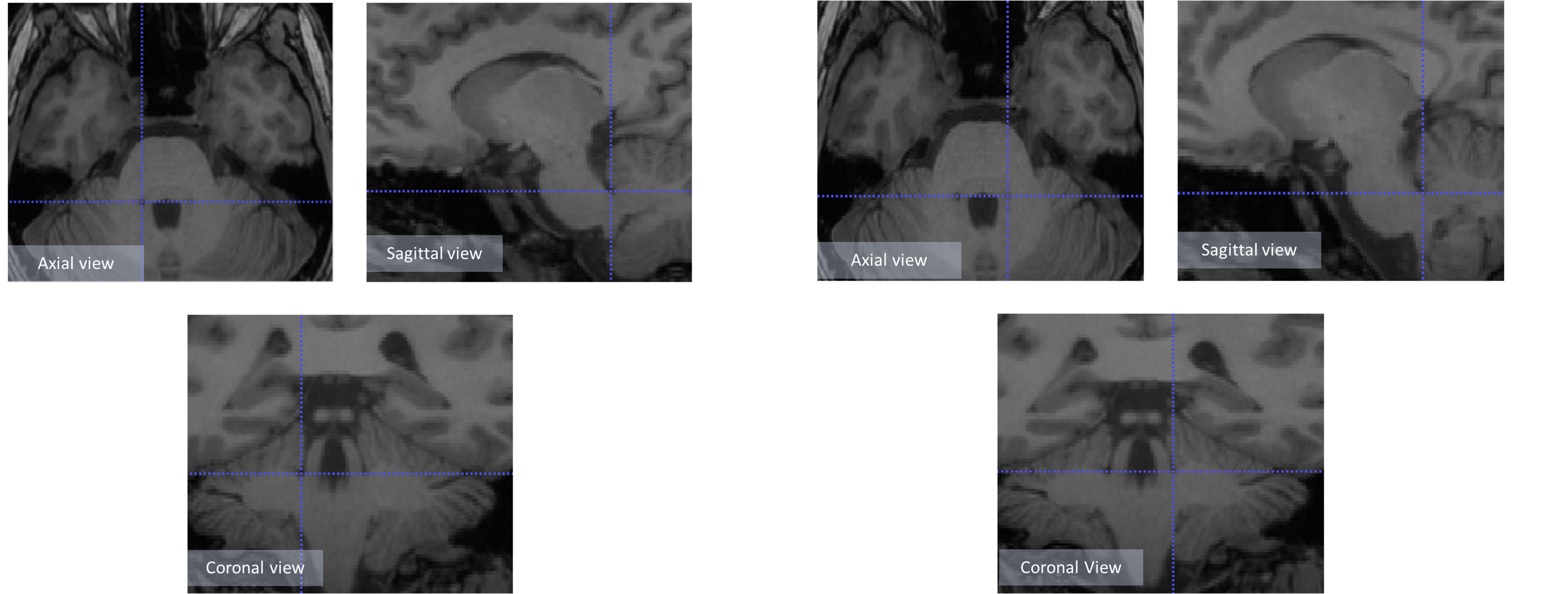


Figure S12: Individuation of two bilateral points employed as anatomical landmarks of the middle cerebellar peduncles
