# Supplementary material for: Fully Automated Segmentation of the Pons and Midbrain Using Human T1 MR Brain Images
Source: PLoS One. 2014 Jan 28;9(1):e85618. doi: 10.1371/journal.pone.0085618 (PMC3904850; doi:10.1371/journal.pone.0085618)
Supplement: Figure S15 — VBM analysis revealed the presence of significant volumetric WM loss in the midbrain and pons of AD patients compared to healthy controls. In order to further validate measurement of brainstem as performed by LABS, we employed voxel-based morphometry (VBM) in order to reveal subtle volumetric loss in AD patients. Data were processed using the SPM8 software where we applied VBM implemented in the VBM8 toolbox, incorporating the DARTEL toolbox that was used to obtain a high-dimensional normalization protocol (http://dbm.neuro.uni-jena.de/vbm.html). Images were bias-corrected, tissue classified, and registered using linear (12-parameter affine) and non-linear transformations, within a unified model. Subsequently, the warped white matter (WM) segment was affine transformed into MNI space and were scaled by the Jacobian determinants of the deformations (modulation). Finally, the modulated volumes were smoothed with a Gaussian kernel of 8 mm. The WM volume maps were statistically analysed using the general linear model based on Gaussian random field theory. We investigated the presence of volumetric differences between AD patients (n°40) and healthy controls (n°40) using unpaired t-test. Age and total intracranial volume (ICV) were included in the model as covariates of no-interest. We selected midbrain and pons as regions of interest (ROIs) for VBM analysis. These ROIs were created with the “aal.02” atlas included in the Wake Forest University Pickatlas software version 1.04 (http://www.fmri.wfubmc.edu/download.htm). Statistical threshold was set at P<0.05 with Family-Wise error (FWE) correction for multiple comparisons within ROIs. As showed in Figure S15, we detected abnormal volumetric losses of the midbrain (PFWE = 0.03; T-value; 3.45; x: 19; y:−18; z:−19) and pons (PFWE = 0.01; T-value; 4.02; x: 10; y:−24; z:−46) in AD patients when compared to age-/sex-matched healthy controls. (DOCX) [file pone.0085618.s015.docx]

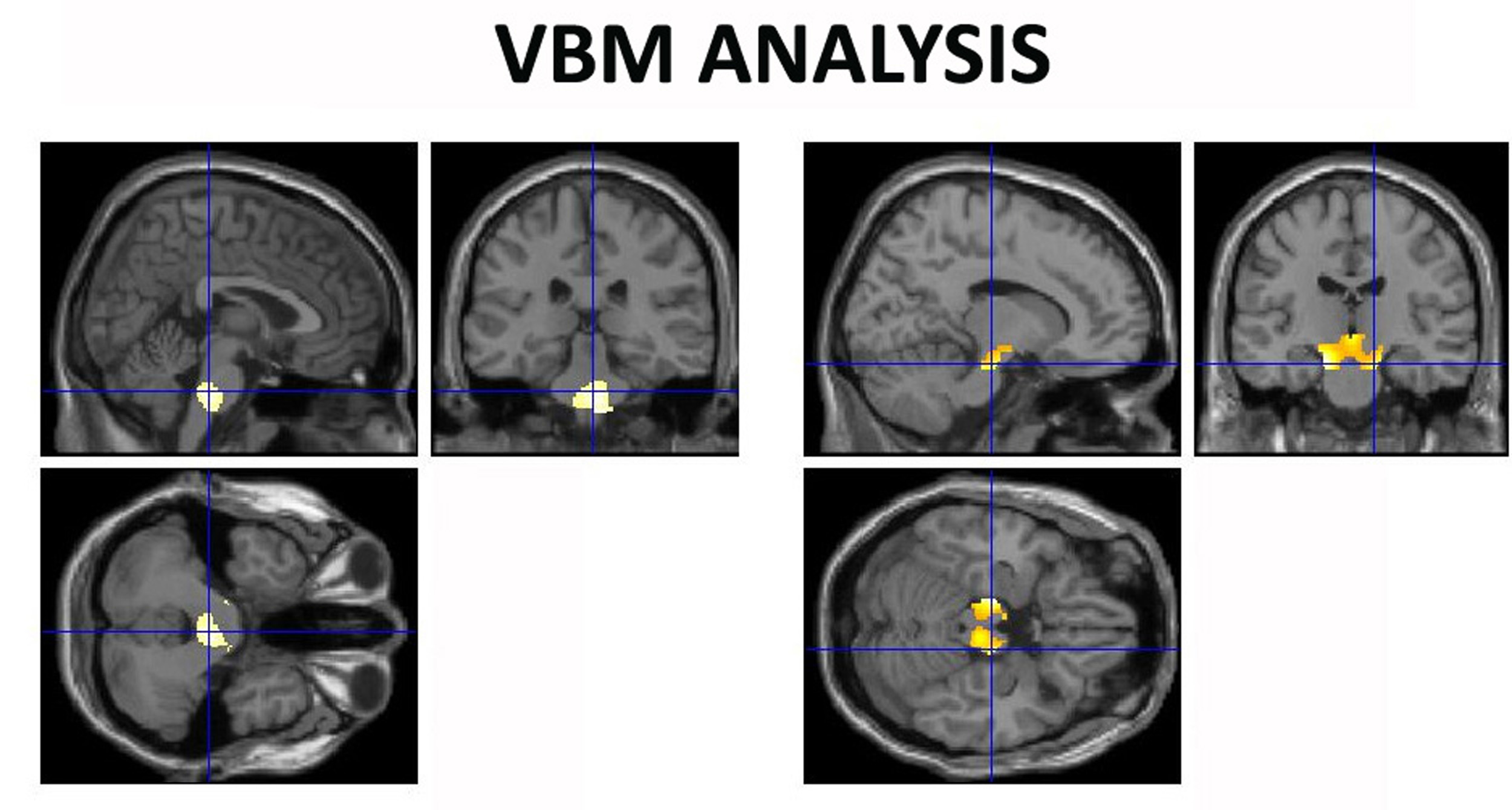


**Figure S15**: VBM analysis revealed the presence of significant volumetric WM loss in the midbrain and pons of AD patients compared to healthy controls. In order to further validate measurement of brainstem as performed by LABS, we employed voxel-based morphometry (VBM) in order to reveal subtle volumetric loss in AD patients. Data were processed using the SPM8 software where we applied VBM implemented in the VBM8 toolbox, incorporating the DARTEL toolbox that was used to obtain a high-dimensional normalization protocol (http://dbm.neuro.uni-jena.de/vbm.html). Images were bias-corrected, tissue classified, and registered using linear (12-parameter affine) and non-linear transformations, within a unified model. Subsequently, the warped white matter (WM) segment was affine transformed into MNI space and were scaled by the Jacobian determinants of the deformations (modulation). Finally, the modulated volumes were smoothed with a Gaussian kernel of 8 mm. The WM volume maps were statistically analysed using the general linear model based on Gaussian random field theory. We investigated the presence of volumetric differences between AD patients (n°40) and healthy controls (n°40) using unpaired *t*-test. Age and total intracranial volume (ICV) were included in the model as covariates of no-interest. We selected midbrain and pons as regions of interest (ROIs) for VBM analysis. These ROIs were created with the “aal.02” atlas included in the Wake Forest University Pickatlas software version 1.04 (<http://www.fmri.wfubmc.edu/download.htm>). Statistical threshold was set at P < 0.05 with Family-Wise error (FWE) correction for multiple comparisons within ROIs.

As showed in Figure S15, we detected abnormal volumetric losses of the midbrain (P_FWE_= 0.03; T-value; 3.45; x: 19; y:-18; z:-19) and pons (P_FWE_= 0.01; T-value; 4.02; x: 10; y:-24; z:-46) in AD patients when compared to age-/sex-matched healthy controls.
