# Supplementary material for: Fully Automated Segmentation of the Pons and Midbrain Using Human T1 MR Brain Images
Source: PLoS One. 2014 Jan 28;9(1):e85618. doi: 10.1371/journal.pone.0085618 (PMC3904850; doi:10.1371/journal.pone.0085618)
Supplement: Table S1 — List of AD patients from the ADNI database that were included in the study. (DOC) [file pone.0085618.s016.doc]

**Table S1:**  List of AD patients from the ADNI database that were included in the study.

| **SUBJECT ID** | **Age** | **Sex** | **Study UID** | **Series UID** | **Image UID** | **LABS Volume (Midbrain)** | **LABS Volume (Pons)** |
| --- | --- | --- | --- | --- | --- | --- | --- |
| 002_S_0816 | 71 | M | 5304 | S19532 | I25405 | 5378,625 | 10525 |
| 002_S_1018 | 71 | F | 6434 | S24311 | I33831 | 5493,375 | 11203,625 |
| 002_S_5018 | 73 | M | 54411 | S174291 | I346242 | 4588 | 9093,625 |
| 006_S_4546 | 71 | M | 43389 | S142616 | I287994 | 4612,75 | 11130,875 |
| 010_S_0829 | 65 | F | 7017 | S26116 | I38572 | 4852,875 | 8651,25 |
| 010_S_5163 | 67 | M | 59059 | S191475 | I375336 | 5070,5 | 9074,75 |
| 012_S_0689 | 66 | M | 6626 | S24939 | I35973 | 5688 | 10171 |
| 012_S_0712 | 77 | M | 6709 | S25202 | I36655 | 4045,875 | 6794,375 |
| 012_S_0720 | 78 | F | 7191 | S26749 | I41028 | 4543,875 | 11077,375 |
| 018_S_0286 | 67 | M | 5938 | S22614 | I29882 | 5331,5 | 11040,5 |
| 018_S_4733 | 75 | M | 45928 | S151381 | I306069 | 4951,625 | 10567,125 |
| 018_S_5074 | 75 | F | 56866 | S182436 | I360314 | 4320,75 | 9152,375 |
| 018_S_5240 | 63 | F | 59894 | S196225 | I382181 | 4014,25 | 9017,125 |
| 019_S_4549 | 79 | M | 47822 | S158276 | I317439 | 5170,25 | 9898,875 |
| 019_S_5012 | 77 | M | 54123 | S173252 | I343916 | 4824,75 | 10463,125 |
| 019_S_5019 | 73 | F | 54299 | S173809 | I345563 | 4133,75 | 9866,625 |
| 027_S_1081 | 85 | M | 6761 | S25357 | I37145 | 3044 | 8168 |
| 032_S_1101 | 71 | F | 5982 | S22783 | I30291 | 4509 | 8220 |
| 032_S_1037 | 74 | M | 5749 | S21957 | I28687 | 4231,875 | 8978,125 |
| 033_S_0724 | 79 | M | 4604 | S17337 | I19772 | 3300 | 6890 |
| 033_S_0733 | 59 | F | 4905 | S18048 | I21947 | 3938 | 10618 |
| 068_S_0109 | 70 | F | 2862 | S10771 | I9746 | 4909 | 10112,75 |
| 053_S_5070 | 71 | M | 56477 | S181275 | I357474 | 4826,75 | 9004,875 |
| 053_S_5208 | 71 | M | 59270 | S192574 | I376939 | 4927,75 | 10641,75 |
| 100_S_5106 | 74 | M | 58022 | S187718 | I368412 | 4361,75 | 9780 |
| 123_S_0091 | 67 | M | 4284 | S15897 | I17568 | 3714,375 | 10679,25 |
| 127_S_0844 | 85 | F | 5393 | S19875 | I25984 | 3839 | 6013 |
| 130_S_4589 | 75 | F | 43837 | S144262 | I291219 | 4266,125 | 10867,25 |
| 130_S_4641 | 74 | F | 53779 | S171997 | I342052 | 4704,75 | 8875 |
| 130_S_4971 | 77 | M | 53819 | S172180 | I342338 | 5970,75 | 12000 |
| 130_S_4982 | 58 | F | 53712 | S171800 | I341787 | 5050,375 | 10106,125 |
| 130_S_4990 | 75 | F | 53904 | S172518 | I342911 | 3973,875 | 9390,75 |
| 130_S_4997 | 61 | F | 54664 | S175145 | I347410 | 3007,25 | 8901,625 |
| 130_S_5006 | 68 | F | 54943 | S175869 | I348490 | 4295,625 | 9442,625 |
| 131_S_0457 | 83 | F | 3720 | S13976 | I14561 | 3306 | 8785 |
| 131_S_5138 | 61 | M | 57930 | S187196 | I367565 | 4453,875 | 9843,75 |
| 133_S_1170 | 73 | M | 6537 | S24674 | I35211 | 3092 | 9608 |
| 136_S_0300 | 59 | M | 16029 | S50401 | I106255 | 5420 | 8838 |
| 136_S_0426 | 80 | M | 3898 | S14581 | I15474 | 4305 | 10745 |
| 136_S_4993 | 72 | F | 53844 | S172271 | I342522 | 3999,5 | 9254,125 |

This table lists the IDs of the subjects and the IDs of their T1 images (UID) as well as the series identifier (SID) and the midbrain and pons volumes as calculated by LABS.
